# Comorbidity profile of patients with concurrent diagnoses of asthma and COPD in Germany

**DOI:** 10.1038/s41598-020-74966-1

**Published:** 2020-10-21

**Authors:** Manas K. Akmatov, Tatiana Ermakova, Jakob Holstiege, Annika Steffen, Dominik von Stillfried, Jörg Bätzing

**Affiliations:** 1 Central Research Institute of Ambulatory Health Care in Germany, Berlin, Germany; 2 Weizenbaum Institute for the Networked Society, Berlin, Germany; 3 Fraunhofer Institute for Open Communication Systems (FOKUS), Berlin, Germany

**Keywords:** Diseases, Health care, Medical research, Risk factors

## Abstract

The aim of this study was to estimate the prevalence of concurrent diagnoses of asthma/COPD and examine its full spectrum of comorbid disorders in Germany. We used nationwide outpatient claims data comprising diagnoses of all statutory health insurees (40+ years) in 2017 (N = 40,477,745). The ICD-10 codes J44 (COPD) and J45 (asthma) were used to identify patients. The odds of 1,060 comorbid disorders were examined in a case–control study design. Of all insurees, 4,632,295 (11%) were diagnosed with either asthma or COPD. Of them, 43% had asthma only, 44% COPD only and 13% both diseases. The prevalence of concurrent asthma/COPD was 1.5% with a slightly higher estimate among females than males (1.6% vs. 1.4%) and constant increase by age in both sexes. Comorbid disorders were very common among these patients. 31 disorders were associated with a strong effect size (odds ratio > 10), including other respiratory diseases, but also bacterial (e.g., mycobacteria, including tuberculosis) and fungal infections (e.g., sporotrichosis and aspergillosis). Patients with concurrent asthma/COPD suffer from comorbid disorders involving various body systems, which points to the need of a multidisciplinary care approach. Regular screening for common comorbid disorders may result in better clinical course and prognosis as well as improvement of patients’ quality of life.

## Introduction

Asthma and chronic obstructive pulmonary disease (COPD) are common chronic diseases characterized by airflow obstruction. Some patients may display clinical features of both diseases, the condition called initially the asthma-COPD overlap syndrome (ACOS)^1,2^. However, the term ‘ACOS’ is no longer advised as it contains various clinical phenotypes with possibly different underlying pathophysiological mechanisms. Recently, the Global Initiative for Asthma (GINA) and the Global Initiative for Chronic Obstructive Lung Disease (GOLD) provided a stepwise approach to diagnose the asthma-COPD overlap (further referred to as ACO) and initial treatment strategy^3^. Many questions regarding etiology and underlying mechanisms remain still unanswered. In particular, there is no well-accepted definition of ACO; diagnostic criteria have not yet been developed either. It is still unknown whether ACO represents a distinct phenotypic condition or is just a disease that features clinical characteristics of both diseases. As a consequence, there is uncertainty regarding the actual disease occurrence. Previous studies reported prevalence estimates for ACO among patients with asthma or COPD varying widely from study to study from 15 to 55%^4^. The prevalence variations resulted mainly from different case definitions, but also from other methodological differences across studies. In contrast, there is agreement that patients with ACO exhibit higher morbidity and mortality compared to patients with asthma or COPD only^1^. Patients with ACO have more rapid and severe disease progression including more frequent exacerbations and a higher risk of some comorbid diseases^5,6^. In particular, cardiovascular diseases are more common among ACO patients as compared to individuals without ACO^7^ or patients with asthma or COPD alone^8–10^. This results in greater healthcare utilization and poorer quality of life^8^.

In Germany, research on the epidemiology of ACO is lacking. A recently published systematic review of the global prevalence of ACO reported a pooled estimate of 2.0% based on 27 population-based studies mostly from industrialized countries such as Canada, USA and several European countries^11^. No single study was found in Germany^11^. The aims of this study were thus to estimate the prevalence of concurrent diagnoses of asthma and COPD in the general German population for the first time and examine the full spectrum of comorbid disorders coexistent in these patients using nationwide ambulatory claims data comprising information on all statutory health insured (SHI) adults in Germany. Using a case–control study design we compared the odds of 1060 comorbid disorders in these patients and controls matched by sex, age and region of residence.

## Results

### Prevalence of concurrent diagnoses of asthma/COPD

**Figure 1:**
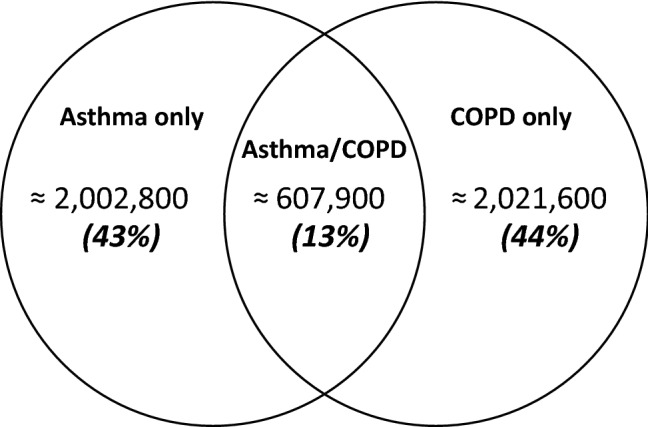
Venn-diagram showing overlap of patients with asthma and COPD diagnoses. COPD, chronic obstructive pulmonary disease.

**Figure 2:**
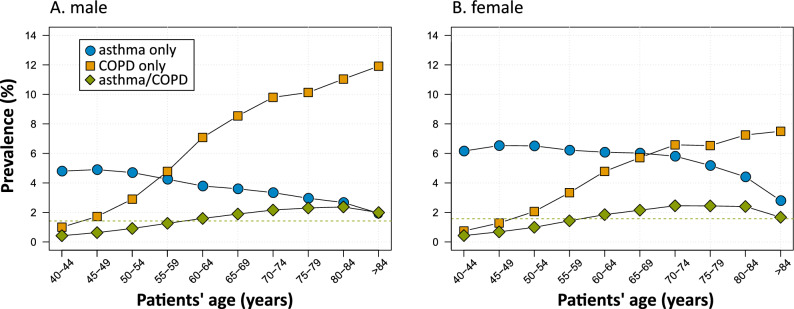
Age-specific prevalence of diagnosis of asthma only, COPD only and concurrent asthma-COPD, stratified by sex, 2017. Dashed lines represent overall prevalence of concurrent asthma/COPD diagnoses (males, 1.57%; females, 1.42%). COPD, chronic obstructive pulmonary disease.

Of the 40,477,745 SHI-individuals, 4,632,295 (11%) were diagnosed with either asthma or COPD. Of them, 43% had asthma only and 44% COPD only (Fig. 1). The remaining 13% had both diagnoses. Among them, the proportion of female patients was higher than male patients (Table 1). The proportion of concurrent diagnoses among patients with asthma was 23.3%. On the other way around, the proportion of concurrent diagnoses among patients with COPD was 23.1%. The crude prevalence of concurrent diagnoses of asthma/COPD in the whole SHI-population was 1.50% (99% CI: 1.50–1.51%). The prevalence was slightly higher among females (1.57%) than males (1.42%) and increased by age in both sexes (Fig. 2); the lowest prevalence was observed among individuals in the youngest age group of 40–45 years in both sexes (males, 0.42%; females, 0.43%). It then increased almost linearly with advancing age reaching the peak in the age group of 80–84 years among males (2.37%) and 70–74 years among females (2.45%). The age-specific prevalence of concurrent asthma/COPD showed a distinctive pattern as compared to that of asthma or COPD only (Fig. 2). Whereas the prevalence of asthma decreased with advancing age, the prevalence of COPD increased (with a more prominent increase among males than females). The weighted prevalence estimate of concurrent asthma/COPD in the total German population ≥ 40 years was 1.39%.

**Table 1:** Selected demographic characteristics of the study and control population.

Characteristics	Cases with concurrent asthma/COPD diagnoses		Controls^a^	
	Number of cases (N = 607,964)	Percent (%)	Number of controls (N = 5,471,676)	Percent (%)
**Sex**				
Male	252,272	41.5	2,270,448	41.5
Female	355,692	58.5	3,201,228	58.5
**Age groups (years)**				
40–49	48,370	8.0	435,330	8.0
50–59	126,967	20.9	1,142,703	20.9
60–69	158,232	26.9	1,424,088	26.9
70–79	164,476	27.1	1,480,284	27.1
80–89	98,340	16.2	885,060	16.2
≥ 90	11,579	1.9	104,211	1.9
**Regional associations of statutory health insurance physicians (ASHIP)**				
Baden-Württemberg	49,695	8.2	447,255	8.2
Bavaria	81,397	13.4	732,573	13.4
Berlin	32,582	5.4	293,238	5.4
Brandenburg	23,911	3.9	215,199	3.9
Bremen	4,669	0.8	42,021	0.8
Hamburg	14,201	2.3	127,809	2.3
Hesse	44,387	7.3	399,483	7.3
Mecklenburg-Western Pomerania	11,615	1.9	104,535	1.9
Lower Saxony	64,819	10.7	583,371	10.7
North Rhine	79,403	13.1	714,627	13.1
Westphalia-Lippe	69,427	11.4	624,843	11.4
Rhineland-Palatinate	30,113	5.0	271,017	5.0
Saarland	7,891	1.3	71,019	1.3
Saxony	23,757	4.6	252,369	4.6
Saxony-Anhalt	18,552	3.1	166,968	3.1
Schleswig–Holstein	23,504	3.9	211,536	3.9
Thuringia	23,757	3.9	213,813	3.9

^a^Control group was matched by sex, age and ASHIP of residence with a case-to-control ratio of 1:9.

### Ranking of comorbid disorders in patients with concurrent asthma/COPD

**Figure 3:**
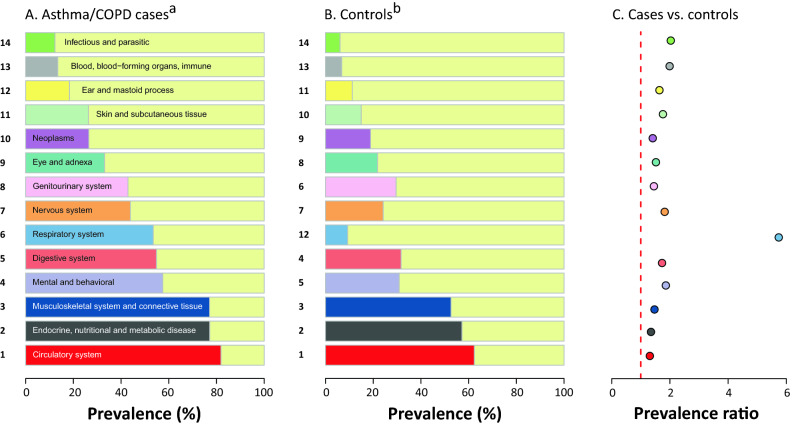
(Co)morbid disorders among adults of age 40 and older with and without asthma/COPD diagnoses, by disease group, 2017. ^a^Ranked by the prevalence of comorbid diseases in patients with concurrent diagnoses of asthma/COPD. ^b^The number on the y axis represents ranking in the control group. Control group was matched by sex, age and ASHIP of residence. A vertical dashed red line in panel C represents a prevalence ratio of 1. ASHIP, regional Associations of Statutory Health Insurance Physicians.

The most prevalent disease groups among patients with concurrent asthma/COPD were diseases of the circulatory system (82%), followed by metabolic and musculoskeletal diseases (each 77%, Fig. 3). In addition, more than 50% of patients had mental, gastrointestinal or other pulmonary diseases. The prevalence of diseases in the control group is presented in Fig. 3B. The ranking of diseases in the control group yielded a similar pattern. Remarkably, all 14 disease groups examined were more prevalent among patients with asthma/COPD than in controls with the prevalence ratios (PR) ranging between 1.31 (99% CI: 1.31–1.31) (circulatory system) and 2.03 (99% CI: 2.01–2.05) (infectious and parasitic diseases) (Fig. 3C). As expected, other pulmonary diseases were more frequently observed among patients with asthma/COPD than in the control group (PR, 5.74; 99% CI: 5.71–5.77).

The top 20 specific comorbid disorders (at the level of the first three characters of the ICD-10 code) among patients with asthma/COPD are presented in Table 2. More than two-thirds of these patients suffered from primary hypertension, every fourth patient had a diagnosis of chronic ischemic heart disease and every sixth patient had chronic heart failure. The most prevalent metabolic disorders were disorders of lipoprotein metabolism and other lipidemias (43%), type 2 diabetes (38%), obesity (27%) and nontoxic goiter (17%). Every fourth patient with asthma/COPD had a diagnosis of depressive episode and every fifth patient had sleep disorders. All 20 top ranked comorbid diseases were more prevalent among patients with asthma/COPD than among controls.

**Table 2:** The top 20 most prevalent comorbid diseases among adults of age 40 and older with concurrent diagnoses of asthma/COPD, based on the first three characters of an ICD-10 code, 2017^a^.

Disease/disorder	ICD-10 code	Cases with concurrent asthma/COPD diagnoses			Controls^b^		
		Rank among cases	Number (N = 607,964)	Prevalence (%)	Rank among controls^c^	Number (N = 5,471,676)	Prevalence (%)
Essential (primary) hypertension	I10	1	414,126	68.1	1	2,808,371	51.3
Disorders of lipoprotein metabolism and other lipidemias	E78	2	262,079	43.1	2	1,758,985	32.1
Dorsalgia	M54	3	231,776	38.1	3	1,138,187	20.8
Type 2 diabetes mellitus	E11	4	168,796	27.8	4	983,285	18.0
Obesity	E66	5	164,326	27.0	9	629,713	11.5
Depressive episode	F32	6	149,621	24.6	8	637,751	11.7
Chronic ischemic heart disease	I25	7	145,365	23.9	7	669,381	12.2
Spondylosis	M47	8	137,539	22.6	10	625,460	11.4
Gastro-esophageal reflux disease	K21	9	130,707	21.5	13	467,761	8.5
Gonarthrosis	M17	10	129,731	21.3	6	700,909	12.8
Disorders of refraction and accommodation	H52	11	121,686	20.0	5	760,163	13.9
Sleep disorders	G47	12	112,423	18.5	34	314,834	5.8
Somatoform disorders	F45	13	111,068	18.3	14	445,757	8.1
Vasomotor and allergic rhinitis	J30	14	110,862	18.2	71	159,837	2.9
Mental and behavioral disorders due to use of tobacco	F17	15	104,204	17.1	63	173,854	3.2
Other intervertebral disc disorders	M51	16	102,754	16.9	16	440,008	8.0
Other nontoxic goiter	E04	17	101,023	16.6	11	614,735	11.2
Heart failure	I50	18	95,064	15.6	31	325,980	6.0
Varicose veins of lower extremities	I83	19	91,932	15.1	12	536,624	9.8
Osteoporosis without pathological fracture	M81	20	88,670	14.6	26	368,262	6.7

^a^Ranked by the most prevalent comorbid diseases in patients with concurrent diagnoses of asthma/COPD.

^b^Control group was matched by sex, age and ASHIP of residence with a case-to-control ratio of 1:9.

^c^The numbers represent the rank of diseases in the control group. ASHIP, regional Associations of Statutory health Insurance Physicians.

### Comorbidity profile

**Figure 4:**
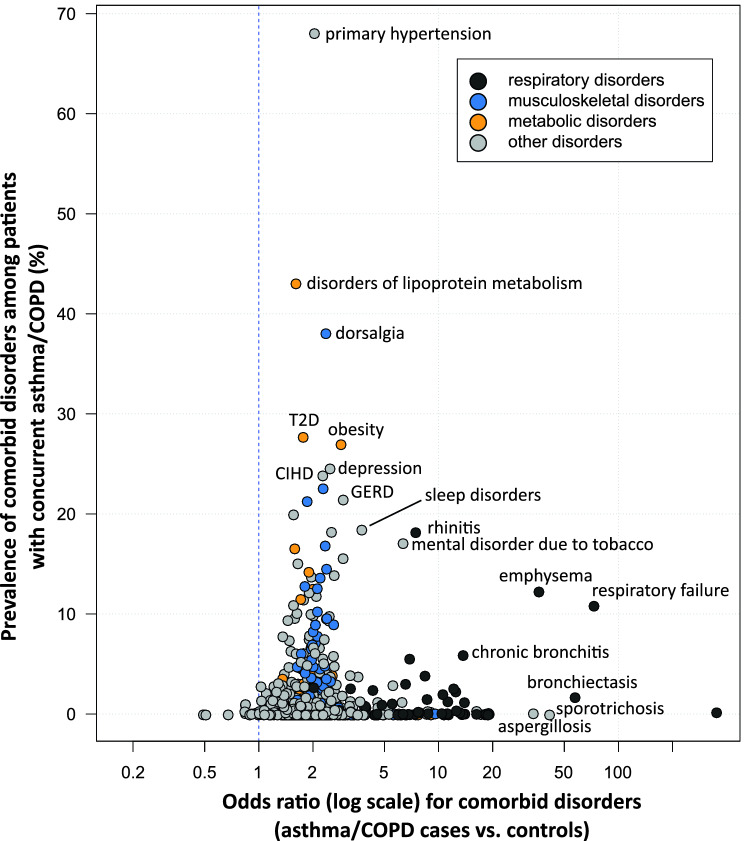
Scatter plot depicting odds ratios for comorbid disorders among patients with concurrent asthma/COPD diagnoses over their prevalence estimates. Each circle represents a comorbid disorder defined by the first three characters of an ICD-10 code. In total, 940 comorbid disorders are depicted; 120 disorders had very low prevalence and could not be depicted due to data protection regulations (n < 30 either in cases or controls). Control group was matched by sex, age and ASHIP of residence. ASHIP, regional Associations of Statutory Health Insurance Physicians; COPD, chronic obstructive pulmonary disease; GERD, gastroesophageal reflux disease; CIHD, chronic ischemic heart disease; T2D, type 2 diabetes.

Out of the total 1079 three-character ICD-10 codes from 14 chapters, we identified 1064 codes among patients with asthma/COPD diagnoses and 1069 codes in the control population, with an overlap of 1060 codes found in both groups. Of the 1060 comorbid disorders examined, 987 (93%) disorders were significantly different among patients with asthma/COPD as compared to controls at the significance level of 5%. After Bonferroni correction, 879 (83%) remained significant (p ≤ 4.72 × 10^–5^). Figure 4 depicts a scatter plot of the odds ratios for comorbid disorders among patients with asthma/COPD over their prevalence estimates. In general, the odds of comorbid disorders were higher among patients than among controls. Comorbid disorders with significant OR < 1 were observed for seven disorders (Alzheimer disease and associated dementia and multiple sclerosis) reflecting survival bias; all other disorders were positively associated with concurrent asthma/COPD. Of the latter, associations can be roughly divided into three groups; those with a small (OR 1.0–1.5), moderate (OR 1.5–5.0) and large size effect (OR > 10). There were 99 disorders associated with asthma/COPD with a small size effect, for most of them the prevalence was very low (< 1%). The majority of comorbid disorders were associated with a medium size effect. Of them, 35 disorders had a prevalence of > 10% (including cardiovascular [e.g., primary hypertension, chronic ischemic heart disease and heart failure], metabolic [disorders of lipoprotein metabolism, type2 diabetes, obesity and nontoxic goiter], and mental disorders [depression and sleep disorders]). Thirty-one disorders had a strong size effect (OR > 10), but most of them were low prevalent (< 1%). As expected, this group mostly included other respiratory disorders. Two respiratory disorders with OR > 10 had prevalence over 10% (respiratory failure [11%] and emphysema [12%]). In addition, patients with concurrent asthma/COPD were more likely to have bacterial (e.g., mycobacteria, including tuberculosis) and fungal infections (e.g., sporotrichosis and aspergillosis).

## Discussion

We made use of the nationwide outpatient claims data comprising information of 86% of the general German population to estimate the prevalence of concurrent diagnoses of asthma/COPD and examine its comorbid disorders. To the best of our knowledge, this is the first epidemiological study to provide robust morbidity estimates of concurrent asthma/COPD in Germany. This is surprising since these patients represent a specific patient population with a greater disease burden as compared to individuals with asthma or COPD only^8,12^.

We observed that 1.50% of the general population was affected by concurrent diseases, corresponding to the absolute number of approximately 608,000 SHI-patients in Germany. The post-stratification weighting—which aims to adjust for possible demographic differences between the study and the source population and thus to provide a nationally representative estimate—resulted in a slightly lower prevalence of 1.39%. Recently, Hosseini et al. reported a global pooled prevalence of ACO in the general population of 2.0% (95% CI: 1.4–2.6%) with the lowest and highest estimates of 0.3% (the UK) and 5.0% (Australia), respectively^11^. The pooled estimate was based on 27 studies from mostly industrialized countries involving primarily adult study participants from 12 European studies (none from Germany). Thus, our weighted estimate of 1.39% is slightly lower than the pooled estimate reported by Hosseini et al., but lies within the 95% confidence interval. It is also in a good agreement with large-scale studies from Canada (1.6%, study participants aged 30+ years)^13^, Denmark (1.2%, 50–64 years)^14^, South Korea (1.4%, 40+ years)^15^ and the Netherlands (1.3%, 45–65 years)^16^. In addition, it should be noticed that our estimate was based on a nationwide population-based sample and can thus be considered highly representative.

According to previous studies, ACO affects on average every third asthma patient and every fourth COPD patient^6^. However, the prevalence estimates from international studies vary as well. In the above mentioned systematic review Hosseini et al. reported ACO estimates in asthma and COPD adult patients ranging between 11 and 51% and between 13 and 55%, respectively^11^. We observed comparable estimates of concurrent asthma/COPD in asthma and COPD patients (about 23%). In other words, every forth patient with asthma and COPD displayed concurrent diagnoses. The large variations in prevalence estimates across studies may be explained by varying case definitions but also by other methodological differences (*e.g.,* study design and involved populations)^16^. A uniform, broadly accepted definition of ACO does not yet exist. A joined endeavor of GINA and GOLD resulted in a clinical description of ACO which involves “persistent airflow limitation with several features usually associated with asthma and several features usually associated with COPD”^2^. A few research groups published case definitions and diagnostic criteria based upon expert consensus which are restricted to certain countries^17,18^. Previous studies used various definitions based on laboratory findings, symptoms or diagnoses^19^. In the current analysis we used nationwide outpatient claims data which contain diagnoses of asthma and COPD coded according to the ICD-10-GM classification^20^. There is no specific ICD-10 code for ACO, and we used a combination of asthma and COPD diagnoses to define an overlap. We applied a very conservative case definition (diagnoses in at least two quarters) to largely exclude false positive cases. This approach is widely accepted in analysis of routine-based claims data and has already been used in studies on asthma and COPD^21,22^ and rather under- than overestimates morbidity. Our approach of defining the overlap is rather indirect and should be considered as approximation to an epidemiological prevalence assessed in a study design with objectively measured parameters. Nevertheless, our prevalence is similar to estimates from other studies which applied case definitions based on objectively measured parameters.

Comorbid disorders in patients with asthma or COPD have been extensively examined. Asthma is associated with allergic diseases such as allergic rhinitis, allergic conjunctivitis and atopic dermatitis^23^. In addition, asthmatics have higher risk of further respiratory, but also cardiovascular and metabolic diseases^23^. Due to the common risk factors (in particular smoking), COPD is associated with bronchial carcinoma and coronary heart disease^24^. Not surprisingly, mental disorders such as depression and anxiety are very common among both, asthma and COPD patients^25,26^. Research on comorbid disorders in patients with ACO is lacking. Existing evidence shows that patients with ACO have a more rapid disease progression than patients with asthma or COPD alone^1^. As a consequence, they have worse quality of life and higher rates of health care utilization. We found a few studies that focused only on specific comorbid disorders. For example, van Boven et al. examined the prevalence for 16 comorbid disorders in a sample of ACO patients treated in primary care in Spain (n = 5093)^27^. The most prevalent comorbid disorders in this study were hypertension (49%), anxiety (38%), diabetes (22%), osteoporosis (19%) and allergic rhinitis (16%). Ding et al. compared selected comorbid disorders in three patient groups (asthma, COPD and ACO) and observed that patients with ACO (n = 523) had higher prevalence of hypertension (56%), elevated cholesterol/hyperlipidemia (29%), arthritis (17%), depression (21%), obesity (13%) and gastroesophageal reflux disease (19%) (compared to asthma patients)^10^. In addition, ACO patients had higher estimates of diabetes (22%) and osteoporosis (11%) as compared to COPD-only patients^10^. In contrast to the above mentioned studies that focused on selected comorbid disorders, we applied a comprehensive exploratory approach by examining a wide spectrum of comorbid disorders (1060 disorders from 14 disease groups). Comorbid disorders were examined at the level of the first three characters of the ICD-10 code, which represent the main disease category. Overall, comorbid disorders were very common in patients with concurrent asthma/COPD as compared to the control group. Notably, 83% of the examined comorbid disorders were still significantly higher in patients with concurrent asthma/COPD after Bonferroni correction. We found disorders involving all body systems, including mental diseases. The most prevalent were disorders of the circulatory system as well as metabolic and musculoskeletal disorders. In addition, every second patient with asthma/COPD suffered from mental, gastrointestinal and other pulmonary diseases.

The large nationwide sample allowed us to identify comorbid disorders with a small size effect (i.e., OR < 1.5), but still significant after correction for multiple testing, which could not be observed in other studies with smaller sample sizes. Common risk factors may explain the coexistence of some diseases (e.g., smoking and bronchial carcinoma). Persistent systemic inflammation in ACO patients may contribute to the development of cardiovascular diseases^28^. Therapy with inhaled corticosteroid may explain the higher risk of osteoporosis as observed in COPD patients^29^, although the evidence is controversial^30^.

### Strengths and limitations

We made use of nationwide claims data comprising information of about 86% of the total German population. Thus, prevalence estimates can be considered highly representative. In addition, data contain all diagnoses made in an outpatient setting which allows to obtain a nearly complete picture of the comorbidity profile of patients with concurrent asthma/COPD. We applied a hypothesis-free approach to examine the full spectrum of comorbid disorders in these patients. These are unique strengths of the study; to the best of our knowledge, such approach has never been done before.

Our study has potential limitations. Our analysis was based on outpatient claims data containing diagnoses coded according to the ICD-10-GM classification. There is no specific code for ACO, instead, we used the codes for asthma (J45) and COPD (J44) to indirectly define an overlap. Objectively measured, clinical and laboratory (e.g., spirometry) data were not available to validate the diagnosis. In addition, a short time period of one year in which the cases were identified might result in misclassification since diagnoses made outside this period were not considered. However, our sample comprised nearly 99% of all SHI-individuals in Germany in 2017. Also, a conservative case definition applied in our study (i.e*.,* at least two diagnoses of each, asthma and COPD, in different quarters of the year) might further contribute to misclassification and result in prevalence underestimation. Thus, some patients, e.g. with well-controlled asthma might not be diagnosed in 2017 even if they visited a physician in that year. On the other hand, claims data are primarily collected for reimbursement purposes and not for morbidity estimation and are known to overestimate the true epidemiological prevalence, in particular, if a case definition is only based on a single diagnosis. Previous research on asthma and COPD based on routine data thus applies a more conservative case definition^21,22,31–33^. Furthermore, per case definition (i.e., diagnoses in at least two different quarters), patients with concurrent asthma/COPD diagnoses have higher rates of health care contacts, in particular, if patients visit different physicians as compared to controls. Thus, the chance of getting other (comorbid) diagnoses may be higher in these patients. In addition, we were restricted to the variables available in our dataset (i.e., sex, age and region of residence). Other potentially confounding variables such as socio-economic status were not available in the data. Finally, it has to be mentioned that some of the associations might still be false positive even after the correction for multiple testing. On the contrary, some real associations may have been overlooked due to the very strict p value.

## Conclusions

This is the first study to provide morbidity estimates of possible ACO in Germany and examine its full spectrum of comorbid disorders. We observed the relatively low prevalence of concurrent asthma/COPD diagnoses at the national level. However, this population group was characterized by a considerable disease burden. (Multi)morbidity is very common in these patients and requires a special attention of researchers and clinicians. Due to the broad comorbidity spectrum, these patients might benefit from a multidisciplinary care approach. Timely identification of comorbid disorders may help to improve their clinical course, prognosis and quality of life.

## Methods

### Data and study population

The present study is based on outpatient claims data received from all regional Associations of Statutory Health Insurance Physicians (ASHIPs) in Germany. In total, there are 17 ASHIPs, of whom 15 coincide with 15 German federal states whereas two ASHIPs exist in the federal state of North Rhine-Westphalia. The data contain diagnoses of all SHI-individuals in Germany who visited a SHI-authorized physician in the year 2017. The diagnoses are coded according to the German modification of the 10th edition of the International Classification of Diseases and Related Health Problems (ICD-10-GM). The study population comprises insurees with available information on sex, age (40+ years) and region of residence (N = 40,477,745). The German population consisted of 47,185,969 inhabitants over 40 years in 2017^34^, 41,062,196 of them were statutory insured^35^. Thus, the study population included approx. 86% of the total German population and nearly 99% of the total SHI-population.

### Case ascertainment and prevalence estimation

We defined a patient with concurrent diagnoses, when each ICD-10 codes J44 ‘other chronic obstructive pulmonary disease’^36,37^ and J45 ‘asthma’^21,31^ were diagnosed in at least two different quarters in 2017, resulting in total of four diagnoses (i.e*.*, two diagnoses of asthma in two different quarters and two COPD diagnoses in two different quarters). In addition, all diagnoses should have a diagnostic modifier “confirmed diagnosis” in contrast to ‘suspected disease’ or ‘cured disease’. The former is usually given for chronic disease cases and for those patients requiring pharmacological therapy^38^.

We first calculated the crude prevalence of concurrent diagnoses in patients with either asthma or COPD and in the whole study SHI-population. For the latter, we applied post-stratification weighting to obtain a nationally representative prevalence estimate. Sex and age (5-year age groups) distribution of the German population from the year 2017 obtained from the Federal Statistical Office^34^ was used to create the respective weights. A post-stratification weighting was only used in the total sample of the SHI-population to estimate a nationally representative prevalence of concurrent asthma/COPD diagnoses.

### Comorbidity profile

Using the case–control study design we compared comorbid disorders among patients with and without concurrent diagnoses (i.e., a control group). A control group was retrieved from a sample of SHI-individuals who also visited a SHI-authorized physician in 2017 for reasons other than asthma and/or COPD. We selected SHI-insurees without a single diagnosis of asthma and/or COPD with available information on sex, age and region of residence (ASHIP). Of them, we only retrieved individuals matched by sex, age and regional ASHIP with the highest case-to-control ratio of 1:9 (n = 5,471,676). For all individuals, i.e., with and without asthma/COPD, we extracted all diagnoses using the procedure mentioned above (i.e., diagnoses with the highest diagnostic certainty “confirmed diagnosis” in at least two quarters of 2017). The ICD-10 consists of 22 chapters representing broad disease categories with codes containing 3–5 characters^20^. In this analysis we used codes with the first three characters, which represent the main disease category (*e.g.,* the code A01 for typhoid and paratyphoid fevers). We included the first 14 chapters of the ICD-10-GM classification, which represent specific diseases (Fig. 3). The remaining chapters of the ICD-10-GM classification were excluded from the further analysis because they represent unspecific symptoms and conditions not directly relevant with regards to asthma/COPD (e.g., injuries, accidents, etc.). We undertook ranking of comorbid disorders among patients with asthma/COPD based on disease prevalence to identify the most relevant comorbid diseases. This was done by disease groups (14 chapters) and diseases based on the first three characters of the ICD-10 code. Furthermore, we compared the prevalence of comorbid disorders in patients with asthma/COPD diagnoses and in the control population separately for each ICD-10 code (at the level of three characters) and for each disease group. We estimated odds ratios (OR) and corresponding 95% confidence intervals (CI) for each of the 1,060 comorbid diseases in patients with and without asthma/COPD. Due to the exploratory character of this analysis, we applied the Bonferroni correction to address the problem of multiple testing (n = 1060 disorders)^39^. A Bonferroni corrected p-value of 4.72 × 10^–5^ (i.e., 0.05/1,060 disorders) was considered statistically significant.

### Data availability

The datasets analysed during the current study are not publicly available due to data protection regulations by the German Social Security Code (Sozialgesetzbuch (SGB) V).

## Supplementary information

Supplementary Information
