# Supplementary material for: Comorbidity profile of patients with concurrent diagnoses of asthma and COPD in Germany
Source: Sci Rep. 2020 Oct 21;10:17945. doi: 10.1038/s41598-020-74966-1 (PMC7578650; doi:10.1038/s41598-020-74966-1)
Supplement: Supplementary file 1 — Supplementary Information [file 41598_2020_74966_MOESM1_ESM.pdf]

Comorbidity profile of patients with concurrent diagnoses of asthma and COPD in Germany

Manas K. Akmatov[1*,+], Tatiana Ermakova[1,2,+], Jakob Holstiege[1], Annika Steffen[1], Dominik von Stillfried[1], Jörg Bätzing[1]

[1] Central Research Institute of Ambulatory Health Care in Germany, Berlin, Germany

[2] Weizenbaum Institute for the Networked Society, Berlin, Germany

[*] makmatov@zi.de

[+] These authors contributed equally to this work.

Supplementary table. Odds ratios, 95% confidence intervals and Bonferroni-corrected p values for the chance of comorbid diseases in patients with concurrent diagnoses of asthma and COPD as compared to matched controls*

Disease group	ICD-10 code	Odds ratio	Lower 95% confidence interval	Upper 95% confidence interval	Bonferroni corrected p value
Certain infectious and parasitic diseases	A00	2.80	1.68	4.69	0.097
Certain infectious and parasitic diseases	A01	1.85	1.26	2.70	1
Certain infectious and parasitic diseases	A02	3.29	2.64	4.09	$<1.0 \times 10^{-13}$
Certain infectious and parasitic diseases	A03	3.07	1.68	5.64	0.320
Certain infectious and parasitic diseases	A04	2.09	1.91	2.29	$<1.0 \times 10^{-13}$
Certain infectious and parasitic diseases	A05	2.62	1.50	4.57	0.785
Certain infectious and parasitic diseases	A06	3.24	2.75	3.81	$<1.0 \times 10^{-13}$
Certain infectious and parasitic diseases	A07	3.00	2.29	3.92	1.24×10^{-12}
Certain infectious and parasitic diseases	A08	2.29	2.09	2.51	$<1.0 \times 10^{-13}$
Certain infectious and parasitic diseases	A09	2.18	2.11	2.24	$<1.0 \times 10^{-13}$
Certain infectious and parasitic diseases	A15	8.54	7.49	9.73	$<1.0 \times 10^{-13}$
Certain infectious and parasitic diseases	A16	8.96	8.38	9.57	$<1.0 \times 10^{-13}$
Certain infectious and parasitic diseases	A17	3.47	2.12	5.68	0.0008
Certain infectious and parasitic diseases	A18	2.51	2.21	2.86	$<1.0 \times 10^{-13}$
Certain infectious and parasitic diseases	A19	2.52	1.39	4.56	1
Certain infectious and parasitic diseases	A20	18.00	1.63	198.52	1
Certain infectious and parasitic diseases	A21	2.35	0.96	5.77	1
Certain infectious and parasitic diseases	A22	7.71	2.59	22.95	0.269
Certain infectious and parasitic diseases	A23	3.38	2.56	4.45	$<1.0 \times 10^{-13}$
Certain infectious and parasitic diseases	A24	8.40	4.05	17.40	1.14×10^{-5}
Certain infectious and parasitic diseases	A25	1.50	0.18	12.46	1
Certain infectious and parasitic diseases	A26	1.41	1.23	1.62	0.0006
Certain infectious and parasitic diseases	A27	1.80	0.62	5.27	1
Certain infectious and parasitic diseases	A28	2.44	2.00	2.99	$<1.0 \times 10^{-13}$
Certain infectious and parasitic diseases	A30	1.17	0.35	3.91	1
Certain infectious and parasitic diseases	A31	17.07	14.32	20.34	$<1.0 \times 10^{-13}$
Certain infectious and parasitic diseases	A32	0.73	0.22	2.37	1
Certain infectious and parasitic diseases	A35	5.79	2.50	13.37	0.044
Certain infectious and parasitic diseases	A36	2.81	1.58	5.02	0.528
Certain infectious and parasitic diseases	A37	6.66	5.67	7.83	$<1.0 \times 10^{-13}$
Certain infectious and parasitic diseases	A38	4.56	3.13	6.63	2.73×10^{-12}
Certain infectious and parasitic diseases	A39	2.35	1.38	4.01	1
Certain infectious and parasitic diseases	A40	2.75	2.12	3.57	4.44×10^{-11}
Certain infectious and parasitic diseases	A41	2.18	2.00	2.37	$<1.0 \times 10^{-13}$
Certain infectious and parasitic diseases	A42	3.27	2.28	4.70	1.51×10^{-7}
Certain infectious and parasitic diseases	A43	5.06	2.24	11.46	0.111
Certain infectious and parasitic diseases	A44	1.38	0.31	6.14	1
Certain infectious and parasitic diseases	A46	2.42	2.32	2.52	$<1.0 \times 10^{-13}$
Certain infectious and parasitic diseases	A48	2.26	1.92	2.67	$<1.0 \times 10^{-13}$

Disease group	ICD-10 code	Odds ratio	Lower 95% confidence interval	Upper 95% confidence interval	Bonferroni corrected p value
Certain infectious and parasitic diseases	A49	2.65	2.54	2.77	$<1.0 \times 10^{-13}$
Certain infectious and parasitic diseases	A50	2.82	2.18	3.64	3.72×10^{-12}
Certain infectious and parasitic diseases	A51	2.45	1.89	3.16	7.27×10^{-9}
Certain infectious and parasitic diseases	A52	2.02	1.84	2.22	$<1.0 \times 10^{-13}$
Certain infectious and parasitic diseases	A53	1.92	1.42	2.61	0.030
Certain infectious and parasitic diseases	A54	3.20	2.63	3.90	$<1.0 \times 10^{-13}$
Certain infectious and parasitic diseases	A55	2.25	0.98	5.15	1
Certain infectious and parasitic diseases	A56	1.78	1.28	2.49	0.804
Certain infectious and parasitic diseases	A57	1.91	1.07	3.40	1
Certain infectious and parasitic diseases	A59	1.90	1.36	2.66	0.205
Certain infectious and parasitic diseases	A60	1.68	1.48	1.90	2.48×10^{-13}
Certain infectious and parasitic diseases	A63	2.24	2.03	2.47	$<1.0 \times 10^{-13}$
Certain infectious and parasitic diseases	A64	1.47	0.83	2.58	1
Certain infectious and parasitic diseases	A66	3.23	1.75	5.95	0.187
Certain infectious and parasitic diseases	A67	3.41	1.71	6.83	0.587
Certain infectious and parasitic diseases	A68	1.47	1.33	1.63	3.22×10^{-11}
Certain infectious and parasitic diseases	A69	1.44	1.39	1.50	$<1.0 \times 10^{-13}$
Certain infectious and parasitic diseases	A70	6.43	3.62	11.41	2.32×10^{-7}
Certain infectious and parasitic diseases	A71	4.50	1.54	13.17	1
Certain infectious and parasitic diseases	A74	3.44	2.99	3.97	$<1.0 \times 10^{-13}$
Certain infectious and parasitic diseases	A75	3.00	0.81	11.08	1
Certain infectious and parasitic diseases	A77	1.55	0.60	4.01	1
Certain infectious and parasitic diseases	A78	1.73	0.66	4.51	1
Certain infectious and parasitic diseases	A79	1.24	0.44	3.53	1
Certain infectious and parasitic diseases	A80	1.54	1.25	1.90	0.058
Certain infectious and parasitic diseases	A81	1.65	1.31	2.07	0.018
Certain infectious and parasitic diseases	A82	3.00	0.31	28.84	1
Certain infectious and parasitic diseases	A83	3.00	0.81	11.08	1
Certain infectious and parasitic diseases	A84	1.84	1.23	2.74	1
Certain infectious and parasitic diseases	A85	1.50	0.63	3.56	1
Certain infectious and parasitic diseases	A86	2.09	1.29	3.40	1
Certain infectious and parasitic diseases	A87	3.71	2.35	5.87	2.02×10^{-5}
Certain infectious and parasitic diseases	A88	1.67	1.35	2.07	0.002
Certain infectious and parasitic diseases	A89	2.25	0.48	10.60	1
Certain infectious and parasitic diseases	A92	1.50	0.18	12.46	1
Certain infectious and parasitic diseases	A93	1.13	0.14	9.00	1
Certain infectious and parasitic diseases	A94	3.00	0.81	11.08	1
Certain infectious and parasitic diseases	A98	1.74	0.73	4.18	1
Certain infectious and parasitic diseases	B00	2.02	1.94	2.11	$<1.0 \times 10^{-13}$
Certain infectious and parasitic diseases	B01	1.92	1.38	2.66	0.121
Certain infectious and parasitic diseases	B02	2.10	2.04	2.17	$<1.0 \times 10^{-13}$
Certain infectious and parasitic diseases	B03	4.50	0.41	49.63	1
Certain infectious and parasitic diseases	B05	2.86	1.57	5.23	0.677

Disease group	ICD-10 code	Odds ratio	Lower 95% confidence interval	Upper 95% confidence interval	Bonferroni corrected p value
Certain infectious and parasitic diseases	B06	3.12	1.46	6.65	1
Certain infectious and parasitic diseases	B07	1.71	1.65	1.77	$<1.0 \times 10^{-13}$
Certain infectious and parasitic diseases	B08	1.91	1.56	2.32	1.84×10^{-7}
Certain infectious and parasitic diseases	B09	2.92	2.10	4.05	2.00×10^{-7}
Certain infectious and parasitic diseases	B15	1.96	1.77	2.16	$<1.0 \times 10^{-13}$
Certain infectious and parasitic diseases	B16	2.41	2.27	2.56	$<1.0 \times 10^{-13}$
Certain infectious and parasitic diseases	B17	2.73	2.55	2.94	$<1.0 \times 10^{-13}$
Certain infectious and parasitic diseases	B18	2.30	2.22	2.38	$<1.0 \times 10^{-13}$
Certain infectious and parasitic diseases	B19	2.30	1.95	2.71	$<1.0 \times 10^{-13}$
Certain infectious and parasitic diseases	B20	1.76	1.31	2.34	0.151
Certain infectious and parasitic diseases	B21	1.70	1.07	2.71	1
Certain infectious and parasitic diseases	B22	2.09	1.62	2.70	1.49×10^{-5}
Certain infectious and parasitic diseases	B23	1.97	1.72	2.25	$<1.0 \times 10^{-13}$
Certain infectious and parasitic diseases	B24	1.40	1.29	1.53	1.24×10^{-11}
Certain infectious and parasitic diseases	B25	2.80	2.41	3.25	$<1.0 \times 10^{-13}$
Certain infectious and parasitic diseases	B26	2.82	1.73	4.61	0.038
Certain infectious and parasitic diseases	B27	2.46	2.19	2.75	$<1.0 \times 10^{-13}$
Certain infectious and parasitic diseases	B30	1.77	1.49	2.11	8.15×10^{-8}
Certain infectious and parasitic diseases	B33	2.38	1.89	2.98	7.14×10^{-11}
Certain infectious and parasitic diseases	B34	1.65	1.57	1.74	$<1.0 \times 10^{-13}$
Certain infectious and parasitic diseases	B35	1.61	1.59	1.64	$<1.0 \times 10^{-13}$
Certain infectious and parasitic diseases	B36	2.08	2.01	2.16	$<1.0 \times 10^{-13}$
Certain infectious and parasitic diseases	B37	4.55	4.43	4.68	$<1.0 \times 10^{-13}$
Certain infectious and parasitic diseases	B38	4.50	0.82	24.57	1
Certain infectious and parasitic diseases	B39	10.00	4.06	24.61	0.0006
Certain infectious and parasitic diseases	B40	2.70	0.74	9.81	1
Certain infectious and parasitic diseases	B41	4.50	0.41	49.63	1
Certain infectious and parasitic diseases	B42	41.15	23.08	73.37	$<1.0 \times 10^{-13}$
Certain infectious and parasitic diseases	B43	2.81	1.03	7.68	1
Certain infectious and parasitic diseases	B44	33.43	28.78	38.84	$<1.0 \times 10^{-13}$
Certain infectious and parasitic diseases	B45	2.32	1.07	5.05	1
Certain infectious and parasitic diseases	B46	1.93	0.80	4.66	1
Certain infectious and parasitic diseases	B48	2.29	2.02	2.60	$<1.0 \times 10^{-13}$
Certain infectious and parasitic diseases	B49	2.32	2.21	2.44	$<1.0 \times 10^{-13}$
Certain infectious and parasitic diseases	B50	2.01	1.38	2.93	0.331
Certain infectious and parasitic diseases	B51	1.50	0.58	3.87	1
Certain infectious and parasitic diseases	B52	4.50	2.02	10.02	0.256
Certain infectious and parasitic diseases	B53	2.70	0.74	9.81	1
Certain infectious and parasitic diseases	B54	2.13	1.25	3.60	1
Certain infectious and parasitic diseases	B55	1.69	0.49	5.79	1
Certain infectious and parasitic diseases	B56	2.25	0.48	10.60	1
Certain infectious and parasitic diseases	B57	1.50	0.34	6.70	1

Disease group	ICD-10 code	Odds ratio	Lower 95% confidence interval	Upper 95% confidence interval	Bonferroni corrected p value
Certain infectious and parasitic diseases	B58	1.95	1.63	2.34	2.98×10^{-10}
Certain infectious and parasitic diseases	B59	3.04	1.90	4.88	0.004
Certain infectious and parasitic diseases	B60	2.10	0.92	4.78	1
Certain infectious and parasitic diseases	B65	3.35	2.38	4.71	5.08×10^{-9}
Certain infectious and parasitic diseases	B66	3.56	2.09	6.06	0.003
Certain infectious and parasitic diseases	B67	1.76	1.57	1.97	$<1.0 \times 10^{-13}$
Certain infectious and parasitic diseases	B68	3.38	1.57	7.26	1
Certain infectious and parasitic diseases	B69	1.92	1.33	2.79	0.592
Certain infectious and parasitic diseases	B70	7.50	2.29	24.58	0.979
Certain infectious and parasitic diseases	B71	3.38	1.74	6.55	0.364
Certain infectious and parasitic diseases	B74	0.75	0.10	5.77	1
Certain infectious and parasitic diseases	B75	4.50	1.13	17.99	1
Certain infectious and parasitic diseases	B76	4.50	0.82	24.57	1
Certain infectious and parasitic diseases	B77	2.55	1.49	4.37	0.734
Certain infectious and parasitic diseases	B78	4.50	1.54	13.17	1
Certain infectious and parasitic diseases	B79	3.60	1.13	11.48	1
Certain infectious and parasitic diseases	B80	1.58	1.20	2.07	1
Certain infectious and parasitic diseases	B81	3.38	1.96	5.80	0.012
Certain infectious and parasitic diseases	B82	1.69	0.83	3.44	1
Certain infectious and parasitic diseases	B83	1.35	0.94	1.94	1
Certain infectious and parasitic diseases	B85	2.29	1.52	3.44	0.076
Certain infectious and parasitic diseases	B86	1.94	1.71	2.21	$<1.0 \times 10^{-13}$
Certain infectious and parasitic diseases	B87	1.80	0.39	8.22	1
Certain infectious and parasitic diseases	B88	2.62	2.16	3.19	$<1.0 \times 10^{-13}$
Certain infectious and parasitic diseases	B89	1.84	1.11	3.06	1
Certain infectious and parasitic diseases	B90	16.16	15.07	17.32	$<1.0 \times 10^{-13}$
Certain infectious and parasitic diseases	B91	1.23	1.12	1.35	0.029
Certain infectious and parasitic diseases	B94	2.07	1.86	2.30	$<1.0 \times 10^{-13}$
Certain infectious and parasitic diseases	B95	2.23	2.00	2.48	$<1.0 \times 10^{-13}$
Certain infectious and parasitic diseases	B96	2.28	2.16	2.42	$<1.0 \times 10^{-13}$
Certain infectious and parasitic diseases	B97	1.83	1.49	2.24	6.94×10^{-6}
Certain infectious and parasitic diseases	B98	2.13	2.03	2.24	$<1.0 \times 10^{-13}$
Certain infectious and parasitic diseases	B99	2.51	2.38	2.64	$<1.0 \times 10^{-13}$
Neoplasms	C00	1.89	1.62	2.20	2.48×10^{-13}
Neoplasms	C01	1.32	1.12	1.55	0.817
Neoplasms	C02	1.53	1.34	1.75	6.96×10^{-7}
Neoplasms	C03	1.53	1.12	2.09	1
Neoplasms	C04	1.72	1.51	1.97	9.92×10^{-13}
Neoplasms	C05	1.67	1.30	2.15	0.083
Neoplasms	C06	1.59	1.32	1.91	0.0007
Neoplasms	C07	1.49	1.24	1.79	0.028
Neoplasms	C08	1.69	1.42	2.02	5.51×10^{-6}
Neoplasms	C09	1.25	1.09	1.44	1

Disease group	ICD-10 code	Odds ratio	Lower 95% confidence interval	Upper 95% confidence interval	Bonferroni corrected p value
Neoplasms	C10	1.29	1.13	1.47	0.172
Neoplasms	C11	1.34	1.04	1.74	1
Neoplasms	C12	2.52	1.39	4.56	1
Neoplasms	C13	1.67	1.41	1.97	1.30×10^{-6}
Neoplasms	C14	1.76	1.44	2.15	4.40×10^{-5}
Neoplasms	C15	1.71	1.57	1.86	$<1.0 \times 10^{-13}$
Neoplasms	C16	1.26	1.19	1.33	5.46×10^{-12}
Neoplasms	C17	1.19	1.03	1.37	1
Neoplasms	C18	1.28	1.24	1.31	$<1.0 \times 10^{-13}$
Neoplasms	C19	1.27	1.13	1.42	0.063
Neoplasms	C20	1.00	0.96	1.05	1
Neoplasms	C21	1.51	1.33	1.71	6.96×10^{-8}
Neoplasms	C22	1.44	1.31	1.57	1.04×10^{-11}
Neoplasms	C23	0.99	0.78	1.26	1
Neoplasms	C24	1.13	0.96	1.34	1
Neoplasms	C25	1.26	1.16	1.36	1.20×10^{-5}
Neoplasms	C26	1.65	1.53	1.78	$<1.0 \times 10^{-13}$
Neoplasms	C30	1.61	1.21	2.14	1
Neoplasms	C31	1.68	1.31	2.15	0.042
Neoplasms	C32	2.12	1.97	2.28	$<1.0 \times 10^{-13}$
Neoplasms	C33	3.75	1.79	7.84	0.497
Neoplasms	C34	6.22	6.04	6.41	$<1.0 \times 10^{-13}$
Neoplasms	C37	2.17	1.56	3.00	0.004
Neoplasms	C38	1.79	1.53	2.08	1.84×10^{-10}
Neoplasms	C39	6.59	5.12	8.49	$<1.0 \times 10^{-13}$
Neoplasms	C40	1.50	1.13	1.98	1
Neoplasms	C41	1.54	1.34	1.76	1.76×10^{-6}
Neoplasms	C43	1.17	1.13	1.21	$<1.0 \times 10^{-13}$
Neoplasms	C44	1.28	1.26	1.30	$<1.0 \times 10^{-13}$
Neoplasms	C45	1.44	1.17	1.78	0.601
Neoplasms	C46	1.83	1.38	2.44	0.034
Neoplasms	C47	1.97	1.36	2.86	0.367
Neoplasms	C48	1.06	0.89	1.25	1
Neoplasms	C49	1.37	1.26	1.49	7.89×10^{-11}
Neoplasms	C50	1.21	1.19	1.23	$<1.0 \times 10^{-13}$
Neoplasms	C51	1.79	1.64	1.96	$<1.0 \times 10^{-13}$
Neoplasms	C52	1.51	1.20	1.90	0.574
Neoplasms	C53	1.66	1.56	1.76	$<1.0 \times 10^{-13}$
Neoplasms	C54	1.01	0.95	1.07	1
Neoplasms	C55	1.52	1.43	1.63	$<1.0 \times 10^{-13}$
Neoplasms	C56	1.09	1.02	1.16	1
Neoplasms	C57	1.35	1.14	1.61	0.773
Neoplasms	C58	2.37	0.88	6.34	1

**Disease group**	**ICD-10 code**	**Odds ratio**	**Lower 95% confidence interval**	**Upper 95% confidence interval**	**Bonferroni corrected p value**
Neoplasms	C60	1.39	1.16	1.66	0.438
Neoplasms	C61	1.31	1.29	1.34	$<1.0 \times 10^{-13}$
Neoplasms	C62	1.45	1.32	1.58	1.74×10^{-12}
Neoplasms	C63	1.49	1.09	2.03	1
Neoplasms	C64	1.48	1.42	1.53	$<1.0 \times 10^{-13}$
Neoplasms	C65	1.65	1.43	1.90	2.94×10^{-9}
Neoplasms	C66	1.54	1.31	1.81	0.0002
Neoplasms	C67	1.66	1.61	1.70	$<1.0 \times 10^{-13}$
Neoplasms	C68	1.73	1.65	1.81	$<1.0 \times 10^{-13}$
Neoplasms	C69	1.49	1.32	1.67	3.40×10^{-8}
Neoplasms	C70	1.66	1.30	2.11	0.041
Neoplasms	C71	1.01	0.91	1.12	1
Neoplasms	C72	1.39	0.99	1.95	1
Neoplasms	C73	1.62	1.54	1.71	$<1.0 \times 10^{-13}$
Neoplasms	C74	1.60	1.25	2.05	0.247
Neoplasms	C75	1.61	1.39	1.87	4.05×10^{-7}
Neoplasms	C76	1.52	1.39	1.66	$<1.0 \times 10^{-13}$
Neoplasms	C77	1.32	1.26	1.38	$<1.0 \times 10^{-13}$
Neoplasms	C78	1.30	1.25	1.36	$<1.0 \times 10^{-13}$
Neoplasms	C79	1.28	1.24	1.33	$<1.0 \times 10^{-13}$
Neoplasms	C80	1.67	1.62	1.72	$<1.0 \times 10^{-13}$
Neoplasms	C81	1.79	1.65	1.94	$<1.0 \times 10^{-13}$
Neoplasms	C82	1.44	1.32	1.57	9.92×10^{-13}
Neoplasms	C83	1.31	1.22	1.41	2.68×10^{-10}
Neoplasms	C84	1.87	1.66	2.11	$<1.0 \times 10^{-13}$
Neoplasms	C85	1.41	1.36	1.47	$<1.0 \times 10^{-13}$
Neoplasms	C86	2.07	1.46	2.95	0.057
Neoplasms	C88	1.63	1.46	1.83	$<1.0 \times 10^{-13}$
Neoplasms	C90	1.38	1.30	1.47	$<1.0 \times 10^{-13}$
Neoplasms	C91	1.26	1.20	1.32	$<1.0 \times 10^{-13}$
Neoplasms	C92	1.42	1.31	1.54	$<1.0 \times 10^{-13}$
Neoplasms	C93	1.81	1.42	2.31	0.002
Neoplasms	C94	1.96	1.67	2.29	$<1.0 \times 10^{-13}$
Neoplasms	C95	1.97	1.83	2.12	$<1.0 \times 10^{-13}$
Neoplasms	C96	2.16	1.83	2.56	$<1.0 \times 10^{-13}$
Neoplasms	C97	1.71	1.43	2.05	6.28×10^{-6}
Neoplasms	D00	2.15	1.74	2.65	1.12×10^{-9}
Neoplasms	D01	1.62	1.42	1.86	4.55×10^{-9}
Neoplasms	D02	4.87	3.94	6.02	$<1.0 \times 10^{-13}$
Neoplasms	D03	1.25	1.15	1.35	5.78×10^{-5}
Neoplasms	D04	1.29	1.26	1.32	$<1.0 \times 10^{-13}$

Disease group	ICD-10 code	Odds ratio	Lower 95% confidence interval	Upper 95% confidence interval	Bonferroni corrected p value
Neoplasms	D05	1.32	1.23	1.43	6.25×10^{-11}
Neoplasms	D06	1.51	1.31	1.74	1.48×10^{-5}
Neoplasms	D07	1.79	1.69	1.90	$<1.0 \times 10^{-13}$
Neoplasms	D09	1.57	1.46	1.68	$<1.0 \times 10^{-13}$
Neoplasms	D10	2.15	1.83	2.53	$<1.0 \times 10^{-13}$
Neoplasms	D11	2.39	2.13	2.69	$<1.0 \times 10^{-13}$
Neoplasms	D12	1.87	1.83	1.92	$<1.0 \times 10^{-13}$
Neoplasms	D13	2.03	1.95	2.11	$<1.0 \times 10^{-13}$
Neoplasms	D14	5.26	4.67	5.92	$<1.0 \times 10^{-13}$
Neoplasms	D15	3.01	2.55	3.56	$<1.0 \times 10^{-13}$
Neoplasms	D16	2.31	2.13	2.51	$<1.0 \times 10^{-13}$
Neoplasms	D17	1.73	1.68	1.78	$<1.0 \times 10^{-13}$
Neoplasms	D18	1.56	1.52	1.60	$<1.0 \times 10^{-13}$
Neoplasms	D19	1.46	0.87	2.43	1
Neoplasms	D20	1.80	0.84	3.85	1
Neoplasms	D21	1.54	1.48	1.61	$<1.0 \times 10^{-13}$
Neoplasms	D22	1.31	1.29	1.33	$<1.0 \times 10^{-13}$
Neoplasms	D23	1.46	1.41	1.51	$<1.0 \times 10^{-13}$
Neoplasms	D24	1.38	1.27	1.49	9.92×10^{-12}
Neoplasms	D25	1.16	1.13	1.19	$<1.0 \times 10^{-13}$
Neoplasms	D26	1.40	1.21	1.62	0.007
Neoplasms	D27	1.40	1.23	1.58	0.0001
Neoplasms	D28	1.62	1.20	2.18	1
Neoplasms	D29	1.57	1.53	1.61	$<1.0 \times 10^{-13}$
Neoplasms	D30	1.62	1.47	1.78	$<1.0 \times 10^{-13}$
Neoplasms	D31	1.41	1.36	1.47	$<1.0 \times 10^{-13}$
Neoplasms	D32	1.52	1.46	1.59	$<1.0 \times 10^{-13}$
Neoplasms	D33	1.40	1.30	1.51	$<1.0 \times 10^{-13}$
Neoplasms	D34	1.60	1.55	1.66	$<1.0 \times 10^{-13}$
Neoplasms	D35	2.19	2.11	2.28	$<1.0 \times 10^{-13}$
Neoplasms	D36	1.90	1.84	1.96	$<1.0 \times 10^{-13}$
Neoplasms	D37	1.91	1.84	1.99	$<1.0 \times 10^{-13}$
Neoplasms	D38	8.64	8.14	9.17	$<1.0 \times 10^{-13}$
Neoplasms	D39	1.56	1.46	1.67	$<1.0 \times 10^{-13}$
Neoplasms	D40	1.50	1.43	1.58	$<1.0 \times 10^{-13}$
Neoplasms	D41	1.78	1.70	1.86	$<1.0 \times 10^{-13}$
Neoplasms	D42	1.64	1.30	2.09	0.049
Neoplasms	D43	1.48	1.35	1.61	$<1.0 \times 10^{-13}$
Neoplasms	D44	2.21	2.10	2.33	$<1.0 \times 10^{-13}$
Neoplasms	D45	2.53	2.39	2.67	$<1.0 \times 10^{-13}$

Disease group	ICD-10 code	Odds ratio	Lower 95% confidence interval	Upper 95% confidence interval	Bonferroni corrected p value
Neoplasms	D46	1.63	1.53	1.73	$<1.0 \times 10^{-13}$
Neoplasms	D47	1.80	1.75	1.86	$<1.0 \times 10^{-13}$
Neoplasms	D48	1.66	1.61	1.70	$<1.0 \times 10^{-13}$
Diseases of the blood and blood-forming organs, and immune disorders	D50	1.98	1.95	2.01	$<1.0 \times 10^{-13}$
Diseases of the blood and blood-forming organs, and immune disorders	D51	1.49	1.45	1.53	$<1.0 \times 10^{-13}$
Diseases of the blood and blood-forming organs, and immune disorders	D52	1.85	1.75	1.95	$<1.0 \times 10^{-13}$
Diseases of the blood and blood-forming organs, and immune disorders	D53	1.96	1.80	2.14	$<1.0 \times 10^{-13}$
Diseases of the blood and blood-forming organs, and immune disorders	D55	2.33	1.82	2.98	1.73×10^{-8}
Diseases of the blood and blood-forming organs, and immune disorders	D56	2.05	1.89	2.23	$<1.0 \times 10^{-13}$
Diseases of the blood and blood-forming organs, and immune disorders	D57	1.84	1.44	2.36	0.002
Diseases of the blood and blood-forming organs, and immune disorders	D58	1.82	1.59	2.08	$<1.0 \times 10^{-13}$
Diseases of the blood and blood-forming organs, and immune disorders	D59	1.77	1.60	1.96	$<1.0 \times 10^{-13}$
Diseases of the blood and blood-forming organs, and immune disorders	D60	1.93	1.47	2.53	0.002
Diseases of the blood and blood-forming organs, and immune disorders	D61	1.63	1.52	1.76	$<1.0 \times 10^{-13}$
Diseases of the blood and blood-forming organs, and immune disorders	D62	1.92	1.68	2.20	$<1.0 \times 10^{-13}$
Diseases of the blood and blood-forming organs, and immune disorders	D63	1.82	1.76	1.88	$<1.0 \times 10^{-13}$
Diseases of the blood and blood-forming organs, and immune disorders	D64	1.93	1.90	1.96	$<1.0 \times 10^{-13}$
Diseases of the blood and blood-forming organs, and immune disorders	D65	2.36	1.86	3.00	2.00×10^{-9}
Diseases of the blood and blood-forming organs, and immune disorders	D66	2.20	1.92	2.52	$<1.0 \times 10^{-13}$
Diseases of the blood and blood-forming organs, and immune disorders	D67	1.85	1.31	2.59	0.460
Diseases of the blood and blood-forming organs, and immune disorders	D68	2.18	2.14	2.22	$<1.0 \times 10^{-13}$
Diseases of the blood and blood-forming organs, and immune disorders	D69	1.65	1.61	1.70	$<1.0 \times 10^{-13}$
Diseases of the blood and blood-forming organs, and immune disorders	D70	1.05	0.99	1.11	1
Diseases of the blood and blood-forming organs, and immune disorders	D71	1.83	0.96	3.51	1
Diseases of the blood and blood-forming organs, and immune disorders	D72	3.82	3.70	3.94	$<1.0 \times 10^{-13}$
Diseases of the blood and blood-forming organs, and immune disorders	D73	1.89	1.77	2.01	$<1.0 \times 10^{-13}$
Diseases of the blood and blood-forming organs, and immune disorders	D74	1.29	0.29	5.66	1
Diseases of the blood and blood-forming organs, and immune disorders	D75	2.70	2.61	2.79	$<1.0 \times 10^{-13}$
Diseases of the blood and blood-forming organs, and immune disorders	D76	6.09	4.69	7.91	$<1.0 \times 10^{-13}$
Diseases of the blood and blood-forming organs, and immune disorders	D77	4.08	2.52	6.60	1.26×10^{-5}
Diseases of the blood and blood-forming organs, and immune disorders	D80	4.74	4.55	4.95	$<1.0 \times 10^{-13}$
Diseases of the blood and blood-forming organs, and immune disorders	D81	3.06	2.75	3.40	$<1.0 \times 10^{-13}$
Diseases of the blood and blood-forming organs, and immune disorders	D82	7.41	5.86	9.38	$<1.0 \times 10^{-13}$
Diseases of the blood and blood-forming organs, and immune disorders	D83	3.60	3.04	4.25	$<1.0 \times 10^{-13}$
Diseases of the blood and blood-forming organs, and immune disorders	D84	3.80	3.57	4.04	$<1.0 \times 10^{-13}$
Diseases of the blood and blood-forming organs, and immune disorders	D86	4.69	4.53	4.85	$<1.0 \times 10^{-13}$
Diseases of the blood and blood-forming organs, and immune disorders	D89	3.29	3.07	3.52	$<1.0 \times 10^{-13}$
Endocrine, nutritional and metabolic diseases	E00	1.70	1.42	2.03	8.23×10^{-6}
Endocrine, nutritional and metabolic diseases	E01	1.51	1.49	1.54	$<1.0 \times 10^{-13}$
Endocrine, nutritional and metabolic diseases	E02	1.60	1.44	1.78	$<1.0 \times 10^{-13}$

Disease group	ICD-10 code	Odds ratio	Lower 95% confidence interval	Upper 95% confidence interval	Bonferroni corrected p value
Endocrine, nutritional and metabolic diseases	E03	1.70	1.69	1.72	$<1.0 \times 10^{-13}$
Endocrine, nutritional and metabolic diseases	E04	1.57	1.56	1.59	$<1.0 \times 10^{-13}$
Endocrine, nutritional and metabolic diseases	E05	1.73	1.71	1.75	$<1.0 \times 10^{-13}$
Endocrine, nutritional and metabolic diseases	E06	1.35	1.33	1.37	$<1.0 \times 10^{-13}$
Endocrine, nutritional and metabolic diseases	E07	1.76	1.74	1.79	$<1.0 \times 10^{-13}$
Endocrine, nutritional and metabolic diseases	E10	1.90	1.87	1.93	$<1.0 \times 10^{-13}$
Endocrine, nutritional and metabolic diseases	E11	1.75	1.74	1.77	$<1.0 \times 10^{-13}$
Endocrine, nutritional and metabolic diseases	E12	2.04	1.79	2.32	$<1.0 \times 10^{-13}$
Endocrine, nutritional and metabolic diseases	E13	2.15	2.09	2.21	$<1.0 \times 10^{-13}$
Endocrine, nutritional and metabolic diseases	E14	1.99	1.97	2.00	$<1.0 \times 10^{-13}$
Endocrine, nutritional and metabolic diseases	E15	1.97	1.20	3.23	1
Endocrine, nutritional and metabolic diseases	E16	2.31	2.18	2.44	$<1.0 \times 10^{-13}$
Endocrine, nutritional and metabolic diseases	E20	1.80	1.67	1.95	$<1.0 \times 10^{-13}$
Endocrine, nutritional and metabolic diseases	E21	2.10	2.05	2.16	$<1.0 \times 10^{-13}$
Endocrine, nutritional and metabolic diseases	E22	1.85	1.69	2.03	$<1.0 \times 10^{-13}$
Endocrine, nutritional and metabolic diseases	E23	2.19	2.06	2.33	$<1.0 \times 10^{-13}$
Endocrine, nutritional and metabolic diseases	E24	7.68	7.11	8.30	$<1.0 \times 10^{-13}$
Endocrine, nutritional and metabolic diseases	E25	2.63	2.26	3.06	$<1.0 \times 10^{-13}$
Endocrine, nutritional and metabolic diseases	E26	1.71	1.53	1.91	$<1.0 \times 10^{-13}$
Endocrine, nutritional and metabolic diseases	E27	3.29	3.11	3.48	$<1.0 \times 10^{-13}$
Endocrine, nutritional and metabolic diseases	E28	1.52	1.46	1.58	$<1.0 \times 10^{-13}$
Endocrine, nutritional and metabolic diseases	E29	2.28	2.19	2.38	$<1.0 \times 10^{-13}$
Endocrine, nutritional and metabolic diseases	E30	5.00	1.68	14.92	1
Endocrine, nutritional and metabolic diseases	E31	3.86	3.34	4.46	$<1.0 \times 10^{-13}$
Endocrine, nutritional and metabolic diseases	E32	7.64	5.98	9.77	$<1.0 \times 10^{-13}$
Endocrine, nutritional and metabolic diseases	E34	1.69	1.64	1.74	$<1.0 \times 10^{-13}$
Endocrine, nutritional and metabolic diseases	E35	1.96	1.52	2.53	0.0003
Endocrine, nutritional and metabolic diseases	E41	2.53	2.26	2.84	$<1.0 \times 10^{-13}$
Endocrine, nutritional and metabolic diseases	E43	1.75	1.39	2.20	0.002
Endocrine, nutritional and metabolic diseases	E44	1.68	1.36	2.08	0.002
Endocrine, nutritional and metabolic diseases	E45	1.96	0.74	5.15	1
Endocrine, nutritional and metabolic diseases	E46	1.91	1.77	2.06	$<1.0 \times 10^{-13}$
Endocrine, nutritional and metabolic diseases	E50	2.10	1.72	2.56	1.81×10^{-10}
Endocrine, nutritional and metabolic diseases	E51	1.30	1.14	1.49	0.102
Endocrine, nutritional and metabolic diseases	E52	4.89	3.16	7.58	1.24×10^{-9}
Endocrine, nutritional and metabolic diseases	E53	1.67	1.63	1.70	$<1.0 \times 10^{-13}$
Endocrine, nutritional and metabolic diseases	E54	2.12	1.50	2.99	0.022
Endocrine, nutritional and metabolic diseases	E55	2.03	2.00	2.06	$<1.0 \times 10^{-13}$
Endocrine, nutritional and metabolic diseases	E56	1.79	1.71	1.88	$<1.0 \times 10^{-13}$
Endocrine, nutritional and metabolic diseases	E58	2.34	2.09	2.62	$<1.0 \times 10^{-13}$

**Disease group**	**ICD-10 code**	**Odds ratio**	**Lower 95% confidence interval**	**Upper 95% confidence interval**	**Bonferroni corrected p value**
Endocrine, nutritional and metabolic diseases	E59	2.41	2.05	2.83	$<1.0 \times 10^{-13}$
Endocrine, nutritional and metabolic diseases	E60	2.01	1.73	2.34	$<1.0 \times 10^{-13}$
Endocrine, nutritional and metabolic diseases	E61	2.12	2.06	2.18	$<1.0 \times 10^{-13}$
Endocrine, nutritional and metabolic diseases	E63	1.79	1.54	2.08	2.93×10^{-11}
Endocrine, nutritional and metabolic diseases	E64	1.92	1.64	2.26	9.92×10^{-13}
Endocrine, nutritional and metabolic diseases	E65	3.21	3.02	3.40	$<1.0 \times 10^{-13}$
Endocrine, nutritional and metabolic diseases	E66	2.85	2.83	2.87	$<1.0 \times 10^{-13}$
Endocrine, nutritional and metabolic diseases	E67	3.20	3.02	3.40	$<1.0 \times 10^{-13}$
Endocrine, nutritional and metabolic diseases	E68	2.96	2.82	3.11	$<1.0 \times 10^{-13}$
Endocrine, nutritional and metabolic diseases	E70	2.17	1.87	2.53	$<1.0 \times 10^{-13}$
Endocrine, nutritional and metabolic diseases	E71	1.63	1.57	1.68	$<1.0 \times 10^{-13}$
Endocrine, nutritional and metabolic diseases	E72	1.86	1.76	1.97	$<1.0 \times 10^{-13}$
Endocrine, nutritional and metabolic diseases	E73	2.46	2.40	2.51	$<1.0 \times 10^{-13}$
Endocrine, nutritional and metabolic diseases	E74	2.14	2.06	2.22	$<1.0 \times 10^{-13}$
Endocrine, nutritional and metabolic diseases	E75	2.04	1.88	2.21	$<1.0 \times 10^{-13}$
Endocrine, nutritional and metabolic diseases	E76	2.13	1.65	2.75	6.58×10^{-6}
Endocrine, nutritional and metabolic diseases	E77	1.83	1.51	2.23	1.36×10^{-6}
Endocrine, nutritional and metabolic diseases	E78	1.60	1.59	1.61	$<1.0 \times 10^{-13}$
Endocrine, nutritional and metabolic diseases	E79	1.89	1.87	1.90	$<1.0 \times 10^{-13}$
Endocrine, nutritional and metabolic diseases	E80	1.08	1.01	1.15	1
Endocrine, nutritional and metabolic diseases	E83	1.76	1.71	1.81	$<1.0 \times 10^{-13}$
Endocrine, nutritional and metabolic diseases	E84	9.00	6.94	11.68	$<1.0 \times 10^{-13}$
Endocrine, nutritional and metabolic diseases	E85	1.80	1.60	2.02	$<1.0 \times 10^{-13}$
Endocrine, nutritional and metabolic diseases	E86	1.39	1.30	1.48	$<1.0 \times 10^{-13}$
Endocrine, nutritional and metabolic diseases	E87	2.25	2.21	2.30	$<1.0 \times 10^{-13}$
Endocrine, nutritional and metabolic diseases	E88	2.55	2.51	2.59	$<1.0 \times 10^{-13}$
Endocrine, nutritional and metabolic diseases	E89	1.47	1.44	1.49	$<1.0 \times 10^{-13}$
Endocrine, nutritional and metabolic diseases	E90	3.10	2.54	3.79	$<1.0 \times 10^{-13}$
Mental and behavioural disorders	F00	0.84	0.81	0.86	$<1.0 \times 10^{-13}$
Mental and behavioural disorders	F01	1.15	1.13	1.18	$<1.0 \times 10^{-13}$
Mental and behavioural disorders	F02	0.84	0.78	0.90	0.0005
Mental and behavioural disorders	F03	1.02	1.01	1.04	1
Mental and behavioural disorders	F04	1.35	1.11	1.66	1
Mental and behavioural disorders	F05	0.98	0.90	1.07	1
Mental and behavioural disorders	F06	1.36	1.34	1.39	$<1.0 \times 10^{-13}$
Mental and behavioural disorders	F07	1.14	1.10	1.18	2.23×10^{-12}
Mental and behavioural disorders	F09	1.79	1.64	1.96	$<1.0 \times 10^{-13}$
Mental and behavioural disorders	F10	2.49	2.45	2.52	$<1.0 \times 10^{-13}$
Mental and behavioural disorders	F11	5.19	4.95	5.43	$<1.0 \times 10^{-13}$
Mental and behavioural disorders	F12	5.60	5.11	6.13	$<1.0 \times 10^{-13}$

Disease group	ICD-10 code	Odds ratio	Lower 95% confidence interval	Upper 95% confidence interval	Bonferroni corrected p value
Mental and behavioural disorders	F13	2.84	2.75	2.94	<1.0 x 10^{-13}
Mental and behavioural disorders	F14	6.89	5.80	8.17	<1.0 x 10^{-13}
Mental and behavioural disorders	F15	5.96	5.42	6.56	<1.0 x 10^{-13}
Mental and behavioural disorders	F16	4.50	3.19	6.34	<1.0 x 10^{-13}
Mental and behavioural disorders	F17	6.30	6.25	6.36	<1.0 x 10^{-13}
Mental and behavioural disorders	F18	3.86	2.61	5.70	1.37 x 10^{-8}
Mental and behavioural disorders	F19	4.56	4.40	4.73	<1.0 x 10^{-13}
Mental and behavioural disorders	F20	1.36	1.32	1.40	<1.0 x 10^{-13}
Mental and behavioural disorders	F21	2.42	2.13	2.76	<1.0 x 10^{-13}
Mental and behavioural disorders	F22	1.43	1.36	1.50	<1.0 x 10^{-13}
Mental and behavioural disorders	F23	2.21	2.06	2.37	<1.0 x 10^{-13}
Mental and behavioural disorders	F24	1.51	0.91	2.53	1
Mental and behavioural disorders	F25	1.73	1.65	1.81	<1.0 x 10^{-13}
Mental and behavioural disorders	F28	1.46	1.27	1.69	0.0001
Mental and behavioural disorders	F29	1.92	1.85	1.99	<1.0 x 10^{-13}
Mental and behavioural disorders	F30	1.85	1.66	2.05	<1.0 x 10^{-13}
Mental and behavioural disorders	F31	1.70	1.64	1.77	<1.0 x 10^{-13}
Mental and behavioural disorders	F32	2.47	2.46	2.49	<1.0 x 10^{-13}
Mental and behavioural disorders	F33	2.44	2.42	2.46	<1.0 x 10^{-13}
Mental and behavioural disorders	F34	2.35	2.31	2.39	<1.0 x 10^{-13}
Mental and behavioural disorders	F38	3.41	3.09	3.76	<1.0 x 10^{-13}
Mental and behavioural disorders	F39	2.25	2.04	2.48	<1.0 x 10^{-13}
Mental and behavioural disorders	F40	2.66	2.60	2.72	<1.0 x 10^{-13}
Mental and behavioural disorders	F41	2.45	2.43	2.47	<1.0 x 10^{-13}
Mental and behavioural disorders	F42	1.84	1.75	1.93	<1.0 x 10^{-13}
Mental and behavioural disorders	F43	2.16	2.14	2.19	<1.0 x 10^{-13}
Mental and behavioural disorders	F44	2.87	2.74	3.01	<1.0 x 10^{-13}
Mental and behavioural disorders	F45	2.52	2.50	2.54	<1.0 x 10^{-13}
Mental and behavioural disorders	F48	2.10	2.07	2.14	<1.0 x 10^{-13}
Mental and behavioural disorders	F50	3.18	3.07	3.30	<1.0 x 10^{-13}
Mental and behavioural disorders	F51	2.25	2.20	2.30	<1.0 x 10^{-13}
Mental and behavioural disorders	F52	1.78	1.76	1.81	<1.0 x 10^{-13}
Mental and behavioural disorders	F53	1.17	0.53	2.56	1
Mental and behavioural disorders	F54	3.14	3.03	3.26	<1.0 x 10^{-13}
Mental and behavioural disorders	F55	3.32	3.11	3.55	<1.0 x 10^{-13}
Mental and behavioural disorders	F59	1.58	1.39	1.79	8.66 x 10^{-10}
Mental and behavioural disorders	F60	2.66	2.60	2.72	<1.0 x 10^{-13}
Mental and behavioural disorders	F61	2.96	2.77	3.15	<1.0 x 10^{-13}
Mental and behavioural disorders	F62	3.32	3.22	3.43	<1.0 x 10^{-13}

Disease group	ICD-10 code	Odds ratio	Lower 95% confidence interval	Upper 95% confidence interval	Bonferroni corrected p value
Mental and behavioural disorders	F63	2.96	2.72	3.22	$<1.0 \times 10^{-13}$
Mental and behavioural disorders	F64	2.17	1.81	2.60	$<1.0 \times 10^{-13}$
Mental and behavioural disorders	F65	1.68	1.27	2.22	0.328
Mental and behavioural disorders	F66	1.60	1.38	1.86	4.37×10^{-7}
Mental and behavioural disorders	F68	2.42	2.30	2.56	$<1.0 \times 10^{-13}$
Mental and behavioural disorders	F69	1.72	1.63	1.81	$<1.0 \times 10^{-13}$
Mental and behavioural disorders	F70	1.57	1.48	1.67	$<1.0 \times 10^{-13}$
Mental and behavioural disorders	F71	0.90	0.83	0.98	1
Mental and behavioural disorders	F72	0.49	0.42	0.57	$<1.0 \times 10^{-13}$
Mental and behavioural disorders	F73	0.50	0.39	0.66	0.0007
Mental and behavioural disorders	F74	2.43	1.21	4.89	1
Mental and behavioural disorders	F78	2.05	1.53	2.74	0.001
Mental and behavioural disorders	F79	1.24	1.18	1.30	$<1.0 \times 10^{-13}$
Mental and behavioural disorders	F80	1.42	1.29	1.57	6.65×10^{-10}
Mental and behavioural disorders	F81	3.36	2.97	3.80	$<1.0 \times 10^{-13}$
Mental and behavioural disorders	F82	1.88	1.63	2.17	$<1.0 \times 10^{-13}$
Mental and behavioural disorders	F83	2.39	1.81	3.15	6.46×10^{-7}
Mental and behavioural disorders	F84	0.67	0.56	0.80	0.009
Mental and behavioural disorders	F88	1.80	0.69	4.70	1
Mental and behavioural disorders	F89	1.15	0.81	1.65	1
Mental and behavioural disorders	F90	2.41	2.25	2.59	$<1.0 \times 10^{-13}$
Mental and behavioural disorders	F91	1.79	1.67	1.92	$<1.0 \times 10^{-13}$
Mental and behavioural disorders	F92	2.52	2.20	2.88	$<1.0 \times 10^{-13}$
Mental and behavioural disorders	F93	2.31	1.62	3.30	0.005
Mental and behavioural disorders	F94	2.97	2.20	3.99	7.74×10^{-10}
Mental and behavioural disorders	F95	2.84	2.64	3.06	$<1.0 \times 10^{-13}$
Mental and behavioural disorders	F98	1.96	1.83	2.10	$<1.0 \times 10^{-13}$
Mental and behavioural disorders	F99	2.20	2.12	2.29	$<1.0 \times 10^{-13}$
Diseases of the nervous system	G00	1.74	1.26	2.42	0.951
Diseases of the nervous system	G01	0.39	0.05	2.90	1
Diseases of the nervous system	G02	2.06	0.95	4.43	1
Diseases of the nervous system	G03	1.86	1.57	2.21	1.36×10^{-9}
Diseases of the nervous system	G04	1.34	1.22	1.47	4.57×10^{-7}
Diseases of the nervous system	G05	1.57	1.23	2.01	0.377
Diseases of the nervous system	G06	1.80	1.47	2.22	2.49×10^{-5}
Diseases of the nervous system	G07	0.90	0.12	7.03	1
Diseases of the nervous system	G08	1.73	1.38	2.17	0.003
Diseases of the nervous system	G09	1.54	1.29	1.83	0.001
Diseases of the nervous system	G10	0.88	0.71	1.11	1
Diseases of the nervous system	G11	1.35	1.22	1.48	1.27×10^{-6}
Diseases of the nervous system	G12	1.50	1.35	1.66	4.46×10^{-12}
Diseases of the nervous system	G13	1.57	1.26	1.95	0.057

Disease group	ICD-10 code	Odds ratio	Lower 95% confidence interval	Upper 95% confidence interval	Bonferroni corrected p value
Diseases of the nervous system	G14	1.36	1.18	1.56	0.015
Diseases of the nervous system	G20	1.19	1.16	1.22	$<1.0 \times 10^{-13}$
Diseases of the nervous system	G21	1.21	1.15	1.28	7.05×10^{-10}
Diseases of the nervous system	G22	1.44	1.11	1.87	1
Diseases of the nervous system	G23	1.16	1.03	1.30	1
Diseases of the nervous system	G24	1.67	1.60	1.74	$<1.0 \times 10^{-13}$
Diseases of the nervous system	G25	2.61	2.58	2.65	$<1.0 \times 10^{-13}$
Diseases of the nervous system	G26	2.38	1.66	3.40	0.002
Diseases of the nervous system	G30	0.84	0.82	0.86	$<1.0 \times 10^{-13}$
Diseases of the nervous system	G31	1.55	1.50	1.60	$<1.0 \times 10^{-13}$
Diseases of the nervous system	G32	1.66	1.43	1.94	9.70×10^{-8}
Diseases of the nervous system	G35	0.83	0.79	0.87	1.74×10^{-12}
Diseases of the nervous system	G36	1.47	1.02	2.11	1
Diseases of the nervous system	G37	1.63	1.46	1.82	$<1.0 \times 10^{-13}$
Diseases of the nervous system	G40	1.42	1.39	1.45	$<1.0 \times 10^{-13}$
Diseases of the nervous system	G41	1.26	1.01	1.58	1
Diseases of the nervous system	G43	1.71	1.69	1.73	$<1.0 \times 10^{-13}$
Diseases of the nervous system	G44	2.35	2.30	2.39	$<1.0 \times 10^{-13}$
Diseases of the nervous system	G45	1.82	1.78	1.85	$<1.0 \times 10^{-13}$
Diseases of the nervous system	G46	1.67	1.53	1.83	$<1.0 \times 10^{-13}$
Diseases of the nervous system	G47	3.72	3.69	3.74	$<1.0 \times 10^{-13}$
Diseases of the nervous system	G50	1.97	1.90	2.03	$<1.0 \times 10^{-13}$
Diseases of the nervous system	G51	1.42	1.37	1.48	$<1.0 \times 10^{-13}$
Diseases of the nervous system	G52	2.57	2.36	2.80	$<1.0 \times 10^{-13}$
Diseases of the nervous system	G53	2.19	2.09	2.31	$<1.0 \times 10^{-13}$
Diseases of the nervous system	G54	2.49	2.42	2.56	$<1.0 \times 10^{-13}$
Diseases of the nervous system	G55	2.44	2.40	2.49	$<1.0 \times 10^{-13}$
Diseases of the nervous system	G56	2.28	2.25	2.31	$<1.0 \times 10^{-13}$
Diseases of the nervous system	G57	2.09	2.04	2.14	$<1.0 \times 10^{-13}$
Diseases of the nervous system	G58	3.18	3.08	3.28	$<1.0 \times 10^{-13}$
Diseases of the nervous system	G59	2.10	1.96	2.24	$<1.0 \times 10^{-13}$
Diseases of the nervous system	G60	2.17	2.06	2.28	$<1.0 \times 10^{-13}$
Diseases of the nervous system	G61	2.11	1.98	2.24	$<1.0 \times 10^{-13}$
Diseases of the nervous system	G62	2.30	2.27	2.32	$<1.0 \times 10^{-13}$
Diseases of the nervous system	G63	2.00	1.98	2.02	$<1.0 \times 10^{-13}$
Diseases of the nervous system	G64	3.06	2.79	3.37	$<1.0 \times 10^{-13}$
Diseases of the nervous system	G70	1.98	1.87	2.11	$<1.0 \times 10^{-13}$
Diseases of the nervous system	G71	2.04	1.89	2.19	$<1.0 \times 10^{-13}$
Diseases of the nervous system	G72	2.81	2.63	3.00	$<1.0 \times 10^{-13}$
Diseases of the nervous system	G73	2.49	2.23	2.77	$<1.0 \times 10^{-13}$

Disease group	ICD-10 code	Odds ratio	Lower 95% confidence interval	Upper 95% confidence interval	Bonferroni corrected p value
Diseases of the nervous system	G80	0.91	0.82	1.00	1
Diseases of the nervous system	G81	1.03	1.01	1.05	1
Diseases of the nervous system	G82	1.07	1.02	1.11	1
Diseases of the nervous system	G83	1.72	1.65	1.79	$<1.0 \times 10^{-13}$
Diseases of the nervous system	G90	2.08	1.95	2.22	$<1.0 \times 10^{-13}$
Diseases of the nervous system	G91	1.00	0.93	1.07	1
Diseases of the nervous system	G92	1.58	1.12	2.24	1
Diseases of the nervous system	G93	1.69	1.64	1.73	$<1.0 \times 10^{-13}$
Diseases of the nervous system	G94	1.64	1.14	2.36	1
Diseases of the nervous system	G95	2.36	2.26	2.46	$<1.0 \times 10^{-13}$
Diseases of the nervous system	G96	2.17	1.87	2.51	$<1.0 \times 10^{-13}$
Diseases of the nervous system	G97	1.86	1.53	2.26	6.71×10^{-7}
Diseases of the nervous system	G98	2.18	1.90	2.49	$<1.0 \times 10^{-13}$
Diseases of the nervous system	G99	2.39	2.29	2.49	$<1.0 \times 10^{-13}$
Diseases of the eye and adnexa	H00	1.36	1.29	1.44	$<1.0 \times 10^{-13}$
Diseases of the eye and adnexa	H01	1.65	1.60	1.71	$<1.0 \times 10^{-13}$
Diseases of the eye and adnexa	H02	1.70	1.67	1.74	$<1.0 \times 10^{-13}$
Diseases of the eye and adnexa	H03	1.88	1.44	2.46	0.004
Diseases of the eye and adnexa	H04	1.75	1.73	1.77	$<1.0 \times 10^{-13}$
Diseases of the eye and adnexa	H05	1.97	1.84	2.10	$<1.0 \times 10^{-13}$
Diseases of the eye and adnexa	H06	2.08	1.94	2.24	$<1.0 \times 10^{-13}$
Diseases of the eye and adnexa	H10	3.55	3.49	3.60	$<1.0 \times 10^{-13}$
Diseases of the eye and adnexa	H11	1.46	1.41	1.51	$<1.0 \times 10^{-13}$
Diseases of the eye and adnexa	H13	1.89	1.73	2.06	$<1.0 \times 10^{-13}$
Diseases of the eye and adnexa	H15	1.54	1.37	1.72	2.45×10^{-10}
Diseases of the eye and adnexa	H16	1.71	1.64	1.78	$<1.0 \times 10^{-13}$
Diseases of the eye and adnexa	H17	1.61	1.55	1.67	$<1.0 \times 10^{-13}$
Diseases of the eye and adnexa	H18	1.60	1.56	1.64	$<1.0 \times 10^{-13}$
Diseases of the eye and adnexa	H19	1.76	1.71	1.81	$<1.0 \times 10^{-13}$
Diseases of the eye and adnexa	H20	1.53	1.44	1.63	$<1.0 \times 10^{-13}$
Diseases of the eye and adnexa	H21	1.48	1.40	1.56	$<1.0 \times 10^{-13}$
Diseases of the eye and adnexa	H22	2.40	1.85	3.10	3.50×10^{-8}
Diseases of the eye and adnexa	H25	1.47	1.45	1.48	$<1.0 \times 10^{-13}$
Diseases of the eye and adnexa	H26	1.62	1.61	1.63	$<1.0 \times 10^{-13}$
Diseases of the eye and adnexa	H27	1.64	1.61	1.68	$<1.0 \times 10^{-13}$
Diseases of the eye and adnexa	H28	1.96	1.86	2.07	$<1.0 \times 10^{-13}$
Diseases of the eye and adnexa	H30	1.77	1.59	1.96	$<1.0 \times 10^{-13}$
Diseases of the eye and adnexa	H31	1.57	1.51	1.63	$<1.0 \times 10^{-13}$
Diseases of the eye and adnexa	H32	1.81	1.27	2.59	1
Diseases of the eye and adnexa	H33	1.43	1.39	1.48	$<1.0 \times 10^{-13}$

Disease group	ICD-10 code	Odds ratio	Lower 95% confidence interval	Upper 95% confidence interval	Bonferroni corrected p value
Diseases of the eye and adnexa	H34	1.45	1.40	1.50	$<1.0 \times 10^{-13}$
Diseases of the eye and adnexa	H35	1.57	1.56	1.59	$<1.0 \times 10^{-13}$
Diseases of the eye and adnexa	H36	1.41	1.39	1.44	$<1.0 \times 10^{-13}$
Diseases of the eye and adnexa	H40	1.44	1.43	1.46	$<1.0 \times 10^{-13}$
Diseases of the eye and adnexa	H42	1.72	1.32	2.23	0.064
Diseases of the eye and adnexa	H43	1.51	1.49	1.54	$<1.0 \times 10^{-13}$
Diseases of the eye and adnexa	H44	1.38	1.31	1.45	$<1.0 \times 10^{-13}$
Diseases of the eye and adnexa	H45	1.62	1.19	2.22	1
Diseases of the eye and adnexa	H46	1.42	1.24	1.62	0.0002
Diseases of the eye and adnexa	H47	1.34	1.32	1.37	$<1.0 \times 10^{-13}$
Diseases of the eye and adnexa	H48	1.36	1.25	1.48	3.06×10^{-10}
Diseases of the eye and adnexa	H49	1.48	1.39	1.58	$<1.0 \times 10^{-13}$
Diseases of the eye and adnexa	H50	1.51	1.49	1.54	$<1.0 \times 10^{-13}$
Diseases of the eye and adnexa	H51	1.53	1.39	1.70	$<1.0 \times 10^{-13}$
Diseases of the eye and adnexa	H52	1.55	1.54	1.56	$<1.0 \times 10^{-13}$
Diseases of the eye and adnexa	H53	1.60	1.58	1.62	$<1.0 \times 10^{-13}$
Diseases of the eye and adnexa	H54	1.60	1.57	1.64	$<1.0 \times 10^{-13}$
Diseases of the eye and adnexa	H55	1.46	1.34	1.60	$<1.0 \times 10^{-13}$
Diseases of the eye and adnexa	H57	1.71	1.64	1.77	$<1.0 \times 10^{-13}$
Diseases of the eye and adnexa	H58	1.85	1.67	2.05	$<1.0 \times 10^{-13}$
Diseases of the eye and adnexa	H59	1.52	1.48	1.56	$<1.0 \times 10^{-13}$
Diseases of the ear and mastoid process	H60	1.74	1.71	1.78	$<1.0 \times 10^{-13}$
Diseases of the ear and mastoid process	H61	1.46	1.44	1.48	$<1.0 \times 10^{-13}$
Diseases of the ear and mastoid process	H62	1.94	1.63	2.31	1.07×10^{-10}
Diseases of the ear and mastoid process	H65	2.87	2.73	3.02	$<1.0 \times 10^{-13}$
Diseases of the ear and mastoid process	H66	2.26	2.18	2.35	$<1.0 \times 10^{-13}$
Diseases of the ear and mastoid process	H67	0.78	0.18	3.32	1
Diseases of the ear and mastoid process	H68	2.16	2.02	2.31	$<1.0 \times 10^{-13}$
Diseases of the ear and mastoid process	H69	2.64	2.48	2.80	$<1.0 \times 10^{-13}$
Diseases of the ear and mastoid process	H70	3.26	2.86	3.70	$<1.0 \times 10^{-13}$
Diseases of the ear and mastoid process	H71	1.97	1.81	2.15	$<1.0 \times 10^{-13}$
Diseases of the ear and mastoid process	H72	2.14	2.00	2.29	$<1.0 \times 10^{-13}$
Diseases of the ear and mastoid process	H73	1.70	1.63	1.78	$<1.0 \times 10^{-13}$
Diseases of the ear and mastoid process	H74	1.93	1.79	2.08	$<1.0 \times 10^{-13}$
Diseases of the ear and mastoid process	H75	2.74	1.51	4.98	1
Diseases of the ear and mastoid process	H80	1.31	1.19	1.45	0.0001
Diseases of the ear and mastoid process	H81	1.80	1.77	1.83	$<1.0 \times 10^{-13}$
Diseases of the ear and mastoid process	H82	1.76	1.58	1.95	$<1.0 \times 10^{-13}$
Diseases of the ear and mastoid process	H83	1.99	1.88	2.12	$<1.0 \times 10^{-13}$
Diseases of the ear and mastoid process	H90	1.84	1.82	1.86	$<1.0 \times 10^{-13}$

Disease group	ICD-10 code	Odds ratio	Lower 95% confidence interval	Upper 95% confidence interval	Bonferroni corrected p value
Diseases of the ear and mastoid process	H91	1.76	1.74	1.78	$<1.0 \times 10^{-13}$
Diseases of the ear and mastoid process	H92	2.21	2.09	2.34	$<1.0 \times 10^{-13}$
Diseases of the ear and mastoid process	H93	1.71	1.69	1.73	$<1.0 \times 10^{-13}$
Diseases of the ear and mastoid process	H94	3.41	2.09	5.58	0.001
Diseases of the ear and mastoid process	H95	1.83	1.68	1.99	$<1.0 \times 10^{-13}$
Diseases of the circulatory system	I00	2.52	2.27	2.80	$<1.0 \times 10^{-13}$
Diseases of the circulatory system	I01	2.33	1.85	2.93	5.03×10^{-10}
Diseases of the circulatory system	I02	2.42	1.56	3.76	0.098
Diseases of the circulatory system	I05	2.65	2.57	2.74	$<1.0 \times 10^{-13}$
Diseases of the circulatory system	I06	2.10	1.98	2.24	$<1.0 \times 10^{-13}$
Diseases of the circulatory system	I07	2.14	2.10	2.17	$<1.0 \times 10^{-13}$
Diseases of the circulatory system	I08	2.02	1.96	2.09	$<1.0 \times 10^{-13}$
Diseases of the circulatory system	I09	2.84	2.45	3.28	$<1.0 \times 10^{-13}$
Diseases of the circulatory system	I10	2.03	2.01	2.04	$<1.0 \times 10^{-13}$
Diseases of the circulatory system	I11	2.36	2.34	2.38	$<1.0 \times 10^{-13}$
Diseases of the circulatory system	I12	2.11	2.06	2.16	$<1.0 \times 10^{-13}$
Diseases of the circulatory system	I13	2.38	2.31	2.45	$<1.0 \times 10^{-13}$
Diseases of the circulatory system	I15	1.73	1.68	1.77	$<1.0 \times 10^{-13}$
Diseases of the circulatory system	I20	2.68	2.64	2.73	$<1.0 \times 10^{-13}$
Diseases of the circulatory system	I21	1.90	1.86	1.94	$<1.0 \times 10^{-13}$
Diseases of the circulatory system	I22	2.21	1.95	2.51	$<1.0 \times 10^{-13}$
Diseases of the circulatory system	I23	1.71	1.40	2.08	0.0002
Diseases of the circulatory system	I24	2.39	2.29	2.50	$<1.0 \times 10^{-13}$
Diseases of the circulatory system	I25	2.25	2.24	2.27	$<1.0 \times 10^{-13}$
Diseases of the circulatory system	I26	3.21	3.13	3.29	$<1.0 \times 10^{-13}$
Diseases of the circulatory system	I27	5.53	5.43	5.64	$<1.0 \times 10^{-13}$
Diseases of the circulatory system	I28	6.11	5.29	7.06	$<1.0 \times 10^{-13}$
Diseases of the circulatory system	I30	3.35	2.70	4.17	$<1.0 \times 10^{-13}$
Diseases of the circulatory system	I31	2.78	2.62	2.95	$<1.0 \times 10^{-13}$
Diseases of the circulatory system	I32	3.27	1.85	5.80	0.055
Diseases of the circulatory system	I33	2.11	1.73	2.57	2.35×10^{-10}
Diseases of the circulatory system	I34	1.94	1.92	1.96	$<1.0 \times 10^{-13}$
Diseases of the circulatory system	I35	1.88	1.86	1.91	$<1.0 \times 10^{-13}$
Diseases of the circulatory system	I36	2.33	2.25	2.41	$<1.0 \times 10^{-13}$
Diseases of the circulatory system	I37	1.87	1.76	1.98	$<1.0 \times 10^{-13}$
Diseases of the circulatory system	I38	2.30	2.20	2.41	$<1.0 \times 10^{-13}$
Diseases of the circulatory system	I39	2.22	1.85	2.67	$<1.0 \times 10^{-13}$
Diseases of the circulatory system	I40	2.96	2.56	3.42	$<1.0 \times 10^{-13}$
Diseases of the circulatory system	I41	4.37	3.25	5.87	$<1.0 \times 10^{-13}$

**Disease group**	**ICD-10 code**	**Odds ratio**	**Lower 95% confidence interval**	**Upper 95% confidence interval**	**Bonferroni corrected p value**
Diseases of the circulatory system	I42	2.62	2.57	2.67	$<1.0 \times 10^{-13}$
Diseases of the circulatory system	I43	2.57	2.34	2.83	$<1.0 \times 10^{-13}$
Diseases of the circulatory system	I44	1.64	1.62	1.67	$<1.0 \times 10^{-13}$
Diseases of the circulatory system	I45	1.95	1.91	1.99	$<1.0 \times 10^{-13}$
Diseases of the circulatory system	I46	2.18	1.92	2.47	$<1.0 \times 10^{-13}$
Diseases of the circulatory system	I47	2.08	2.04	2.12	$<1.0 \times 10^{-13}$
Diseases of the circulatory system	I48	1.76	1.75	1.78	$<1.0 \times 10^{-13}$
Diseases of the circulatory system	I49	2.00	1.98	2.02	$<1.0 \times 10^{-13}$
Diseases of the circulatory system	I50	2.93	2.90	2.95	$<1.0 \times 10^{-13}$
Diseases of the circulatory system	I51	2.36	2.32	2.39	$<1.0 \times 10^{-13}$
Diseases of the circulatory system	I52	2.95	2.34	3.72	$<1.0 \times 10^{-13}$
Diseases of the circulatory system	I60	1.25	1.15	1.37	0.0004
Diseases of the circulatory system	I61	0.98	0.92	1.04	1
Diseases of the circulatory system	I62	1.20	1.10	1.31	0.072
Diseases of the circulatory system	I63	1.29	1.27	1.32	$<1.0 \times 10^{-13}$
Diseases of the circulatory system	I64	1.54	1.50	1.57	$<1.0 \times 10^{-13}$
Diseases of the circulatory system	I65	1.80	1.77	1.82	$<1.0 \times 10^{-13}$
Diseases of the circulatory system	I66	1.74	1.64	1.85	$<1.0 \times 10^{-13}$
Diseases of the circulatory system	I67	1.76	1.74	1.78	$<1.0 \times 10^{-13}$
Diseases of the circulatory system	I68	1.48	1.20	1.82	0.244
Diseases of the circulatory system	I69	1.38	1.36	1.40	$<1.0 \times 10^{-13}$
Diseases of the circulatory system	I70	2.07	2.06	2.09	$<1.0 \times 10^{-13}$
Diseases of the circulatory system	I71	1.98	1.94	2.02	$<1.0 \times 10^{-13}$
Diseases of the circulatory system	I72	2.10	2.01	2.19	$<1.0 \times 10^{-13}$
Diseases of the circulatory system	I73	2.26	2.23	2.28	$<1.0 \times 10^{-13}$
Diseases of the circulatory system	I74	2.29	2.20	2.39	$<1.0 \times 10^{-13}$
Diseases of the circulatory system	I77	2.31	2.24	2.38	$<1.0 \times 10^{-13}$
Diseases of the circulatory system	I78	1.59	1.49	1.70	$<1.0 \times 10^{-13}$
Diseases of the circulatory system	I79	2.04	1.99	2.09	$<1.0 \times 10^{-13}$
Diseases of the circulatory system	I80	2.10	2.05	2.14	$<1.0 \times 10^{-13}$
Diseases of the circulatory system	I81	1.42	1.18	1.70	0.174
Diseases of the circulatory system	I82	2.07	2.01	2.12	$<1.0 \times 10^{-13}$
Diseases of the circulatory system	I83	1.64	1.63	1.65	$<1.0 \times 10^{-13}$
Diseases of the circulatory system	I85	1.66	1.57	1.75	$<1.0 \times 10^{-13}$
Diseases of the circulatory system	I86	1.56	1.48	1.65	$<1.0 \times 10^{-13}$
Diseases of the circulatory system	I87	1.89	1.87	1.91	$<1.0 \times 10^{-13}$
Diseases of the circulatory system	I88	2.79	2.57	3.02	$<1.0 \times 10^{-13}$
Diseases of the circulatory system	I89	2.17	2.14	2.20	$<1.0 \times 10^{-13}$
Diseases of the circulatory system	I95	1.82	1.78	1.85	$<1.0 \times 10^{-13}$

**Disease group**	**ICD-10 code**	**Odds ratio**	**Lower 95% confidence interval**	**Upper 95% confidence interval**	**Bonferroni corrected p value**
Diseases of the circulatory system	I97	1.85	1.78	1.92	$<1.0 \times 10^{-13}$
Diseases of the circulatory system	I98	1.50	1.27	1.77	0.002
Diseases of the circulatory system	I99	2.06	2.01	2.11	$<1.0 \times 10^{-13}$
Diseases of the respiratory system	J00	2.51	2.39	2.63	$<1.0 \times 10^{-13}$
Diseases of the respiratory system	J01	3.89	3.76	4.02	$<1.0 \times 10^{-13}$
Diseases of the respiratory system	J02	2.78	2.67	2.90	$<1.0 \times 10^{-13}$
Diseases of the respiratory system	J03	2.59	2.45	2.74	$<1.0 \times 10^{-13}$
Diseases of the respiratory system	J04	3.92	3.78	4.06	$<1.0 \times 10^{-13}$
Diseases of the respiratory system	J05	3.53	2.42	5.13	5.10×10^{-8}
Diseases of the respiratory system	J06	2.01	1.98	2.04	$<1.0 \times 10^{-13}$
Diseases of the respiratory system	J09	4.26	1.93	9.42	0.379
Diseases of the respiratory system	J10	3.80	2.94	4.90	$<1.0 \times 10^{-13}$
Diseases of the respiratory system	J11	3.34	3.09	3.60	$<1.0 \times 10^{-13}$
Diseases of the respiratory system	J12	6.17	4.67	8.16	$<1.0 \times 10^{-13}$
Diseases of the respiratory system	J13	10.65	7.76	14.61	$<1.0 \times 10^{-13}$
Diseases of the respiratory system	J14	7.94	3.97	15.90	5.52×10^{-6}
Diseases of the respiratory system	J15	11.10	10.04	12.28	$<1.0 \times 10^{-13}$
Diseases of the respiratory system	J16	6.48	5.03	8.35	$<1.0 \times 10^{-13}$
Diseases of the respiratory system	J17	5.88	4.46	7.76	$<1.0 \times 10^{-13}$
Diseases of the respiratory system	J18	8.57	8.33	8.81	$<1.0 \times 10^{-13}$
Diseases of the respiratory system	J20	6.51	6.39	6.63	$<1.0 \times 10^{-13}$
Diseases of the respiratory system	J21	18.20	15.81	20.97	$<1.0 \times 10^{-13}$
Diseases of the respiratory system	J22	7.79	7.26	8.35	$<1.0 \times 10^{-13}$
Diseases of the respiratory system	J30	7.41	7.35	7.47	$<1.0 \times 10^{-13}$
Diseases of the respiratory system	J31	4.28	4.20	4.37	$<1.0 \times 10^{-13}$
Diseases of the respiratory system	J32	6.86	6.76	6.96	$<1.0 \times 10^{-13}$
Diseases of the respiratory system	J33	10.49	10.22	10.77	$<1.0 \times 10^{-13}$
Diseases of the respiratory system	J34	3.22	3.16	3.28	$<1.0 \times 10^{-13}$
Diseases of the respiratory system	J35	2.73	2.60	2.87	$<1.0 \times 10^{-13}$
Diseases of the respiratory system	J36	2.48	1.83	3.35	3.85×10^{-6}
Diseases of the respiratory system	J37	5.48	5.32	5.65	$<1.0 \times 10^{-13}$
Diseases of the respiratory system	J38	4.80	4.66	4.96	$<1.0 \times 10^{-13}$
Diseases of the respiratory system	J39	13.83	13.34	14.34	$<1.0 \times 10^{-13}$
Diseases of the respiratory system	J40	8.35	8.20	8.50	$<1.0 \times 10^{-13}$
Diseases of the respiratory system	J41	12.38	12.07	12.70	$<1.0 \times 10^{-13}$
Diseases of the respiratory system	J42	13.60	13.38	13.83	$<1.0 \times 10^{-13}$
Diseases of the respiratory system	J43	35.90	35.35	36.46	$<1.0 \times 10^{-13}$
Diseases of the respiratory system	J46	349.39	252.10	484.23	$<1.0 \times 10^{-13}$
Diseases of the respiratory system	J47	57.05	54.19	60.05	$<1.0 \times 10^{-13}$

Disease group	ICD-10 code	Odds ratio	Lower 95% confidence interval	Upper 95% confidence interval	Bonferroni corrected p value
Diseases of the respiratory system	J60	18.01	14.07	23.03	$<1.0 \times 10^{-13}$
Diseases of the respiratory system	J61	7.66	7.19	8.15	$<1.0 \times 10^{-13}$
Diseases of the respiratory system	J62	10.35	9.39	11.41	$<1.0 \times 10^{-13}$
Diseases of the respiratory system	J63	4.40	3.51	5.52	$<1.0 \times 10^{-13}$
Diseases of the respiratory system	J64	13.43	11.05	16.31	$<1.0 \times 10^{-13}$
Diseases of the respiratory system	J65	11.25	5.83	21.71	5.97×10^{-10}
Diseases of the respiratory system	J66	18.67	11.79	29.55	$<1.0 \times 10^{-13}$
Diseases of the respiratory system	J67	13.06	12.03	14.18	$<1.0 \times 10^{-13}$
Diseases of the respiratory system	J68	18.82	16.88	20.98	$<1.0 \times 10^{-13}$
Diseases of the respiratory system	J69	1.99	1.70	2.32	$<1.0 \times 10^{-13}$
Diseases of the respiratory system	J70	6.75	5.99	7.60	$<1.0 \times 10^{-13}$
Diseases of the respiratory system	J80	16.16	13.99	18.67	$<1.0 \times 10^{-13}$
Diseases of the respiratory system	J81	4.52	4.12	4.98	$<1.0 \times 10^{-13}$
Diseases of the respiratory system	J82	19.00	16.18	22.33	$<1.0 \times 10^{-13}$
Diseases of the respiratory system	J84	11.18	10.82	11.55	$<1.0 \times 10^{-13}$
Diseases of the respiratory system	J85	11.27	8.82	14.40	$<1.0 \times 10^{-13}$
Diseases of the respiratory system	J86	7.36	6.53	8.30	$<1.0 \times 10^{-13}$
Diseases of the respiratory system	J90	3.23	3.07	3.39	$<1.0 \times 10^{-13}$
Diseases of the respiratory system	J91	2.27	1.90	2.71	$<1.0 \times 10^{-13}$
Diseases of the respiratory system	J92	13.90	12.22	15.81	$<1.0 \times 10^{-13}$
Diseases of the respiratory system	J93	8.69	7.92	9.54	$<1.0 \times 10^{-13}$
Diseases of the respiratory system	J94	12.47	11.74	13.25	$<1.0 \times 10^{-13}$
Diseases of the respiratory system	J95	6.30	5.66	7.01	$<1.0 \times 10^{-13}$
Diseases of the respiratory system	J96	72.66	71.07	74.27	$<1.0 \times 10^{-13}$
Diseases of the respiratory system	J98	12.05	11.77	12.34	$<1.0 \times 10^{-13}$
Diseases of the respiratory system	J99	13.56	11.97	15.36	$<1.0 \times 10^{-13}$
Diseases of the digestive system	K00	2.26	1.73	2.94	1.62×10^{-6}
Diseases of the digestive system	K01	0.87	0.42	1.79	1
Diseases of the digestive system	K02	1.81	1.55	2.12	1.18×10^{-10}
Diseases of the digestive system	K03	2.47	1.84	3.32	2.27×10^{-6}
Diseases of the digestive system	K04	2.13	1.85	2.44	$<1.0 \times 10^{-13}$
Diseases of the digestive system	K05	1.72	1.54	1.92	$<1.0 \times 10^{-13}$
Diseases of the digestive system	K06	1.69	1.34	2.14	0.014
Diseases of the digestive system	K07	2.33	2.20	2.46	$<1.0 \times 10^{-13}$
Diseases of the digestive system	K08	1.92	1.72	2.14	$<1.0 \times 10^{-13}$
Diseases of the digestive system	K09	2.43	1.93	3.06	4.81×10^{-11}
Diseases of the digestive system	K10	2.62	2.32	2.95	$<1.0 \times 10^{-13}$
Diseases of the digestive system	K11	2.52	2.38	2.67	$<1.0 \times 10^{-13}$
Diseases of the digestive system	K12	2.69	2.49	2.91	$<1.0 \times 10^{-13}$

**Disease group**	**ICD-10 code**	**Odds ratio**	**Lower 95% confidence interval**	**Upper 95% confidence interval**	**Bonferroni corrected p value**
Diseases of the digestive system	K13	2.77	2.61	2.94	$<1.0 \times 10^{-13}$
Diseases of the digestive system	K14	2.63	2.47	2.80	$<1.0 \times 10^{-13}$
Diseases of the digestive system	K20	2.74	2.63	2.85	$<1.0 \times 10^{-13}$
Diseases of the digestive system	K21	2.93	2.91	2.95	$<1.0 \times 10^{-13}$
Diseases of the digestive system	K22	2.35	2.30	2.40	$<1.0 \times 10^{-13}$
Diseases of the digestive system	K23	2.49	1.61	3.84	0.043
Diseases of the digestive system	K25	2.68	2.61	2.75	$<1.0 \times 10^{-13}$
Diseases of the digestive system	K26	2.47	2.39	2.55	$<1.0 \times 10^{-13}$
Diseases of the digestive system	K27	2.64	2.42	2.87	$<1.0 \times 10^{-13}$
Diseases of the digestive system	K28	2.54	2.28	2.84	$<1.0 \times 10^{-13}$
Diseases of the digestive system	K29	2.61	2.59	2.63	$<1.0 \times 10^{-13}$
Diseases of the digestive system	K30	2.32	2.25	2.39	$<1.0 \times 10^{-13}$
Diseases of the digestive system	K31	2.52	2.47	2.58	$<1.0 \times 10^{-13}$
Diseases of the digestive system	K35	2.28	1.99	2.61	$<1.0 \times 10^{-13}$
Diseases of the digestive system	K36	2.72	2.32	3.19	$<1.0 \times 10^{-13}$
Diseases of the digestive system	K37	2.01	1.86	2.18	$<1.0 \times 10^{-13}$
Diseases of the digestive system	K38	2.14	1.86	2.47	$<1.0 \times 10^{-13}$
Diseases of the digestive system	K40	1.71	1.68	1.75	$<1.0 \times 10^{-13}$
Diseases of the digestive system	K41	2.52	2.16	2.93	$<1.0 \times 10^{-13}$
Diseases of the digestive system	K42	2.54	2.47	2.61	$<1.0 \times 10^{-13}$
Diseases of the digestive system	K43	2.55	2.48	2.62	$<1.0 \times 10^{-13}$
Diseases of the digestive system	K44	2.59	2.56	2.62	$<1.0 \times 10^{-13}$
Diseases of the digestive system	K45	2.72	2.46	3.00	$<1.0 \times 10^{-13}$
Diseases of the digestive system	K46	2.69	2.56	2.84	$<1.0 \times 10^{-13}$
Diseases of the digestive system	K50	1.99	1.92	2.06	$<1.0 \times 10^{-13}$
Diseases of the digestive system	K51	1.89	1.84	1.95	$<1.0 \times 10^{-13}$
Diseases of the digestive system	K52	2.45	2.39	2.51	$<1.0 \times 10^{-13}$
Diseases of the digestive system	K55	2.34	2.20	2.49	$<1.0 \times 10^{-13}$
Diseases of the digestive system	K56	2.23	2.15	2.32	$<1.0 \times 10^{-13}$
Diseases of the digestive system	K57	1.94	1.92	1.95	$<1.0 \times 10^{-13}$
Diseases of the digestive system	K58	2.04	2.00	2.08	$<1.0 \times 10^{-13}$
Diseases of the digestive system	K59	2.16	2.13	2.20	$<1.0 \times 10^{-13}$
Diseases of the digestive system	K60	2.12	2.03	2.21	$<1.0 \times 10^{-13}$
Diseases of the digestive system	K61	2.47	2.21	2.76	$<1.0 \times 10^{-13}$
Diseases of the digestive system	K62	1.95	1.91	2.01	$<1.0 \times 10^{-13}$
Diseases of the digestive system	K63	1.92	1.88	1.95	$<1.0 \times 10^{-13}$
Diseases of the digestive system	K64	1.92	1.89	1.94	$<1.0 \times 10^{-13}$
Diseases of the digestive system	K65	2.15	1.89	2.44	$<1.0 \times 10^{-13}$
Diseases of the digestive system	K66	2.42	2.34	2.51	$<1.0 \times 10^{-13}$

Disease group	ICD-10 code	Odds ratio	Lower 95% confidence interval	Upper 95% confidence interval	Bonferroni corrected p value
Diseases of the digestive system	K67	2.40	1.27	4.54	1
Diseases of the digestive system	K70	2.13	2.06	2.20	$<1.0 \times 10^{-13}$
Diseases of the digestive system	K71	2.02	1.93	2.11	$<1.0 \times 10^{-13}$
Diseases of the digestive system	K72	1.81	1.67	1.96	$<1.0 \times 10^{-13}$
Diseases of the digestive system	K73	2.20	2.08	2.32	$<1.0 \times 10^{-13}$
Diseases of the digestive system	K74	1.89	1.83	1.94	$<1.0 \times 10^{-13}$
Diseases of the digestive system	K75	1.95	1.87	2.04	$<1.0 \times 10^{-13}$
Diseases of the digestive system	K76	1.95	1.93	1.96	$<1.0 \times 10^{-13}$
Diseases of the digestive system	K77	2.10	1.93	2.29	$<1.0 \times 10^{-13}$
Diseases of the digestive system	K80	1.50	1.48	1.52	$<1.0 \times 10^{-13}$
Diseases of the digestive system	K81	1.90	1.81	1.99	$<1.0 \times 10^{-13}$
Diseases of the digestive system	K82	1.65	1.60	1.71	$<1.0 \times 10^{-13}$
Diseases of the digestive system	K83	1.75	1.67	1.83	$<1.0 \times 10^{-13}$
Diseases of the digestive system	K85	2.33	2.23	2.44	$<1.0 \times 10^{-13}$
Diseases of the digestive system	K86	2.20	2.15	2.24	$<1.0 \times 10^{-13}$
Diseases of the digestive system	K87	2.06	1.67	2.55	2.42×10^{-8}
Diseases of the digestive system	K90	2.22	2.13	2.32	$<1.0 \times 10^{-13}$
Diseases of the digestive system	K91	1.79	1.73	1.85	$<1.0 \times 10^{-13}$
Diseases of the digestive system	K92	2.64	2.54	2.75	$<1.0 \times 10^{-13}$
Diseases of the digestive system	K93	2.51	1.92	3.28	2.23×10^{-8}
Diseases of the skin and subcutaneous tissue	L00	3.23	1.91	5.45	0.013
Diseases of the skin and subcutaneous tissue	L01	2.17	1.97	2.39	$<1.0 \times 10^{-13}$
Diseases of the skin and subcutaneous tissue	L02	2.63	2.53	2.74	$<1.0 \times 10^{-13}$
Diseases of the skin and subcutaneous tissue	L03	1.94	1.86	2.03	$<1.0 \times 10^{-13}$
Diseases of the skin and subcutaneous tissue	L04	2.89	2.49	3.35	$<1.0 \times 10^{-13}$
Diseases of the skin and subcutaneous tissue	L05	2.59	2.26	2.97	$<1.0 \times 10^{-13}$
Diseases of the skin and subcutaneous tissue	L08	2.20	2.08	2.32	$<1.0 \times 10^{-13}$
Diseases of the skin and subcutaneous tissue	L10	1.88	1.60	2.21	2.26×10^{-11}
Diseases of the skin and subcutaneous tissue	L11	2.06	1.57	2.71	0.0002
Diseases of the skin and subcutaneous tissue	L12	2.10	1.90	2.32	$<1.0 \times 10^{-13}$
Diseases of the skin and subcutaneous tissue	L13	2.23	1.97	2.53	$<1.0 \times 10^{-13}$
Diseases of the skin and subcutaneous tissue	L14	9.00	1.27	63.89	1
Diseases of the skin and subcutaneous tissue	L20	3.20	3.15	3.25	$<1.0 \times 10^{-13}$
Diseases of the skin and subcutaneous tissue	L21	1.71	1.66	1.76	$<1.0 \times 10^{-13}$
Diseases of the skin and subcutaneous tissue	L22	1.14	0.89	1.46	1
Diseases of the skin and subcutaneous tissue	L23	2.89	2.83	2.95	$<1.0 \times 10^{-13}$
Diseases of the skin and subcutaneous tissue	L24	2.05	1.85	2.28	$<1.0 \times 10^{-13}$
Diseases of the skin and subcutaneous tissue	L25	2.37	2.26	2.49	$<1.0 \times 10^{-13}$
Diseases of the skin and subcutaneous tissue	L26	3.79	2.62	5.48	1.75×10^{-9}
Diseases of the skin and subcutaneous tissue	L27	3.23	3.06	3.40	$<1.0 \times 10^{-13}$

Disease group	ICD-10 code	Odds ratio	Lower 95% confidence interval	Upper 95% confidence interval	Bonferroni corrected p value
Diseases of the skin and subcutaneous tissue	L28	2.73	2.61	2.85	$<1.0 \times 10^{-13}$
Diseases of the skin and subcutaneous tissue	L29	2.51	2.45	2.57	$<1.0 \times 10^{-13}$
Diseases of the skin and subcutaneous tissue	L30	2.04	2.02	2.07	$<1.0 \times 10^{-13}$
Diseases of the skin and subcutaneous tissue	L40	1.85	1.82	1.87	$<1.0 \times 10^{-13}$
Diseases of the skin and subcutaneous tissue	L41	1.71	1.54	1.89	$<1.0 \times 10^{-13}$
Diseases of the skin and subcutaneous tissue	L42	1.62	1.33	1.98	0.002
Diseases of the skin and subcutaneous tissue	L43	1.83	1.73	1.92	$<1.0 \times 10^{-13}$
Diseases of the skin and subcutaneous tissue	L44	2.05	1.70	2.48	7.89×10^{-11}
Diseases of the skin and subcutaneous tissue	L45	2.57	0.53	12.38	1
Diseases of the skin and subcutaneous tissue	L50	3.63	3.54	3.73	$<1.0 \times 10^{-13}$
Diseases of the skin and subcutaneous tissue	L51	2.02	1.66	2.45	1.77×10^{-9}
Diseases of the skin and subcutaneous tissue	L52	2.69	2.34	3.10	$<1.0 \times 10^{-13}$
Diseases of the skin and subcutaneous tissue	L53	1.93	1.73	2.15	$<1.0 \times 10^{-13}$
Diseases of the skin and subcutaneous tissue	L54	3.90	2.03	7.48	0.046
Diseases of the skin and subcutaneous tissue	L55	2.01	1.72	2.35	$<1.0 \times 10^{-13}$
Diseases of the skin and subcutaneous tissue	L56	2.17	2.07	2.27	$<1.0 \times 10^{-13}$
Diseases of the skin and subcutaneous tissue	L57	1.25	1.22	1.27	$<1.0 \times 10^{-13}$
Diseases of the skin and subcutaneous tissue	L58	1.87	1.57	2.22	1.23×10^{-9}
Diseases of the skin and subcutaneous tissue	L59	1.94	1.71	2.20	$<1.0 \times 10^{-13}$
Diseases of the skin and subcutaneous tissue	L60	1.97	1.91	2.04	$<1.0 \times 10^{-13}$
Diseases of the skin and subcutaneous tissue	L62	3.07	1.68	5.64	0.320
Diseases of the skin and subcutaneous tissue	L63	1.79	1.69	1.91	$<1.0 \times 10^{-13}$
Diseases of the skin and subcutaneous tissue	L64	1.53	1.45	1.63	$<1.0 \times 10^{-13}$
Diseases of the skin and subcutaneous tissue	L65	1.68	1.62	1.73	$<1.0 \times 10^{-13}$
Diseases of the skin and subcutaneous tissue	L66	1.07	0.92	1.25	1
Diseases of the skin and subcutaneous tissue	L67	3.53	2.42	5.13	5.10×10^{-8}
Diseases of the skin and subcutaneous tissue	L68	1.84	1.69	1.99	$<1.0 \times 10^{-13}$
Diseases of the skin and subcutaneous tissue	L70	1.88	1.81	1.95	$<1.0 \times 10^{-13}$
Diseases of the skin and subcutaneous tissue	L71	1.38	1.35	1.40	$<1.0 \times 10^{-13}$
Diseases of the skin and subcutaneous tissue	L72	1.57	1.51	1.64	$<1.0 \times 10^{-13}$
Diseases of the skin and subcutaneous tissue	L73	2.20	2.08	2.32	$<1.0 \times 10^{-13}$
Diseases of the skin and subcutaneous tissue	L74	2.54	2.16	2.99	$<1.0 \times 10^{-13}$
Diseases of the skin and subcutaneous tissue	L75	1.81	1.34	2.46	0.135
Diseases of the skin and subcutaneous tissue	L80	1.40	1.32	1.48	$<1.0 \times 10^{-13}$
Diseases of the skin and subcutaneous tissue	L81	1.43	1.38	1.48	$<1.0 \times 10^{-13}$
Diseases of the skin and subcutaneous tissue	L82	1.34	1.31	1.38	$<1.0 \times 10^{-13}$
Diseases of the skin and subcutaneous tissue	L83	2.26	1.60	3.20	0.004
Diseases of the skin and subcutaneous tissue	L84	2.14	2.05	2.23	$<1.0 \times 10^{-13}$
Diseases of the skin and subcutaneous tissue	L85	2.18	2.11	2.24	$<1.0 \times 10^{-13}$
Diseases of the skin and subcutaneous tissue	L86	1.80	0.39	8.22	1

Disease group	ICD-10 code	Odds ratio	Lower 95% confidence interval	Upper 95% confidence interval	Bonferroni corrected p value
Diseases of the skin and subcutaneous tissue	L87	2.23	1.78	2.78	1.85×10^{-9}
Diseases of the skin and subcutaneous tissue	L88	2.07	1.64	2.61	7.79×10^{-7}
Diseases of the skin and subcutaneous tissue	L89	1.37	1.33	1.41	$<1.0 \times 10^{-13}$
Diseases of the skin and subcutaneous tissue	L90	1.78	1.72	1.84	$<1.0 \times 10^{-13}$
Diseases of the skin and subcutaneous tissue	L91	1.75	1.61	1.89	$<1.0 \times 10^{-13}$
Diseases of the skin and subcutaneous tissue	L92	2.05	1.91	2.21	$<1.0 \times 10^{-13}$
Diseases of the skin and subcutaneous tissue	L93	2.28	2.15	2.42	$<1.0 \times 10^{-13}$
Diseases of the skin and subcutaneous tissue	L94	1.69	1.52	1.87	$<1.0 \times 10^{-13}$
Diseases of the skin and subcutaneous tissue	L95	2.35	2.02	2.73	$<1.0 \times 10^{-13}$
Diseases of the skin and subcutaneous tissue	L97	1.70	1.65	1.75	$<1.0 \times 10^{-13}$
Diseases of the skin and subcutaneous tissue	L98	1.85	1.80	1.91	$<1.0 \times 10^{-13}$
Diseases of the skin and subcutaneous tissue	L99	2.76	2.21	3.43	$<1.0 \times 10^{-13}$
Diseases of the musculoskeletal system and connective tissue	M00	2.12	1.91	2.37	$<1.0 \times 10^{-13}$
Diseases of the musculoskeletal system and connective tissue	M01	2.05	1.63	2.59	1.31×10^{-6}
Diseases of the musculoskeletal system and connective tissue	M02	1.99	1.85	2.14	$<1.0 \times 10^{-13}$
Diseases of the musculoskeletal system and connective tissue	M03	3.25	2.40	4.41	4.39×10^{-11}
Diseases of the musculoskeletal system and connective tissue	M05	2.20	2.14	2.26	$<1.0 \times 10^{-13}$
Diseases of the musculoskeletal system and connective tissue	M06	2.31	2.28	2.34	$<1.0 \times 10^{-13}$
Diseases of the musculoskeletal system and connective tissue	M07	2.17	2.09	2.26	$<1.0 \times 10^{-13}$
Diseases of the musculoskeletal system and connective tissue	M08	2.37	2.07	2.70	$<1.0 \times 10^{-13}$
Diseases of the musculoskeletal system and connective tissue	M09	2.34	1.98	2.76	$<1.0 \times 10^{-13}$
Diseases of the musculoskeletal system and connective tissue	M10	1.95	1.93	1.98	$<1.0 \times 10^{-13}$
Diseases of the musculoskeletal system and connective tissue	M11	2.07	1.91	2.23	$<1.0 \times 10^{-13}$
Diseases of the musculoskeletal system and connective tissue	M12	2.19	2.01	2.38	$<1.0 \times 10^{-13}$
Diseases of the musculoskeletal system and connective tissue	M13	2.23	2.19	2.27	$<1.0 \times 10^{-13}$
Diseases of the musculoskeletal system and connective tissue	M14	2.20	2.07	2.35	$<1.0 \times 10^{-13}$
Diseases of the musculoskeletal system and connective tissue	M15	1.99	1.97	2.01	$<1.0 \times 10^{-13}$
Diseases of the musculoskeletal system and connective tissue	M16	1.79	1.78	1.81	$<1.0 \times 10^{-13}$
Diseases of the musculoskeletal system and connective tissue	M17	1.85	1.83	1.86	$<1.0 \times 10^{-13}$
Diseases of the musculoskeletal system and connective tissue	M18	2.09	2.06	2.13	$<1.0 \times 10^{-13}$
Diseases of the musculoskeletal system and connective tissue	M19	2.10	2.08	2.12	$<1.0 \times 10^{-13}$
Diseases of the musculoskeletal system and connective tissue	M20	1.62	1.60	1.65	$<1.0 \times 10^{-13}$
Diseases of the musculoskeletal system and connective tissue	M21	1.71	1.69	1.73	$<1.0 \times 10^{-13}$
Diseases of the musculoskeletal system and connective tissue	M22	1.91	1.87	1.95	$<1.0 \times 10^{-13}$
Diseases of the musculoskeletal system and connective tissue	M23	1.80	1.77	1.82	$<1.0 \times 10^{-13}$
Diseases of the musculoskeletal system and connective tissue	M24	1.84	1.78	1.90	$<1.0 \times 10^{-13}$
Diseases of the musculoskeletal system and connective tissue	M25	2.05	2.03	2.07	$<1.0 \times 10^{-13}$
Diseases of the musculoskeletal system and connective tissue	M30	9.52	8.58	10.57	$<1.0 \times 10^{-13}$
Diseases of the musculoskeletal system and connective tissue	M31	1.91	1.82	2.00	$<1.0 \times 10^{-13}$

Disease group	ICD-10 code	Odds ratio	Lower 95% confidence interval	Upper 95% confidence interval	Bonferroni corrected p value
Diseases of the musculoskeletal system and connective tissue	M32	2.31	2.14	2.49	$<1.0 \times 10^{-13}$
Diseases of the musculoskeletal system and connective tissue	M33	2.36	2.11	2.65	$<1.0 \times 10^{-13}$
Diseases of the musculoskeletal system and connective tissue	M34	1.96	1.83	2.11	$<1.0 \times 10^{-13}$
Diseases of the musculoskeletal system and connective tissue	M35	1.94	1.91	1.97	$<1.0 \times 10^{-13}$
Diseases of the musculoskeletal system and connective tissue	M36	3.30	2.53	4.32	$<1.0 \times 10^{-13}$
Diseases of the musculoskeletal system and connective tissue	M40	2.27	2.24	2.31	$<1.0 \times 10^{-13}$
Diseases of the musculoskeletal system and connective tissue	M41	1.95	1.93	1.97	$<1.0 \times 10^{-13}$
Diseases of the musculoskeletal system and connective tissue	M42	2.10	2.09	2.12	$<1.0 \times 10^{-13}$
Diseases of the musculoskeletal system and connective tissue	M43	2.17	2.15	2.20	$<1.0 \times 10^{-13}$
Diseases of the musculoskeletal system and connective tissue	M45	1.77	1.72	1.84	$<1.0 \times 10^{-13}$
Diseases of the musculoskeletal system and connective tissue	M46	2.40	2.31	2.49	$<1.0 \times 10^{-13}$
Diseases of the musculoskeletal system and connective tissue	M47	2.27	2.25	2.28	$<1.0 \times 10^{-13}$
Diseases of the musculoskeletal system and connective tissue	M48	2.38	2.35	2.40	$<1.0 \times 10^{-13}$
Diseases of the musculoskeletal system and connective tissue	M49	2.79	2.59	3.00	$<1.0 \times 10^{-13}$
Diseases of the musculoskeletal system and connective tissue	M50	2.36	2.32	2.40	$<1.0 \times 10^{-13}$
Diseases of the musculoskeletal system and connective tissue	M51	2.33	2.31	2.34	$<1.0 \times 10^{-13}$
Diseases of the musculoskeletal system and connective tissue	M53	2.18	2.16	2.20	$<1.0 \times 10^{-13}$
Diseases of the musculoskeletal system and connective tissue	M54	2.35	2.33	2.36	$<1.0 \times 10^{-13}$
Diseases of the musculoskeletal system and connective tissue	M60	2.43	2.18	2.71	$<1.0 \times 10^{-13}$
Diseases of the musculoskeletal system and connective tissue	M61	2.04	1.73	2.40	$<1.0 \times 10^{-13}$
Diseases of the musculoskeletal system and connective tissue	M62	1.99	1.97	2.02	$<1.0 \times 10^{-13}$
Diseases of the musculoskeletal system and connective tissue	M63	1.99	1.62	2.43	3.01×10^{-8}
Diseases of the musculoskeletal system and connective tissue	M65	1.90	1.87	1.94	$<1.0 \times 10^{-13}$
Diseases of the musculoskeletal system and connective tissue	M66	2.38	2.13	2.66	$<1.0 \times 10^{-13}$
Diseases of the musculoskeletal system and connective tissue	M67	1.86	1.80	1.92	$<1.0 \times 10^{-13}$
Diseases of the musculoskeletal system and connective tissue	M68	2.29	1.50	3.50	0.133
Diseases of the musculoskeletal system and connective tissue	M70	2.20	2.15	2.26	$<1.0 \times 10^{-13}$
Diseases of the musculoskeletal system and connective tissue	M71	1.93	1.88	1.99	$<1.0 \times 10^{-13}$
Diseases of the musculoskeletal system and connective tissue	M72	1.63	1.59	1.67	$<1.0 \times 10^{-13}$
Diseases of the musculoskeletal system and connective tissue	M73	5.56	4.27	7.25	$<1.0 \times 10^{-13}$
Diseases of the musculoskeletal system and connective tissue	M75	2.05	2.03	2.07	$<1.0 \times 10^{-13}$
Diseases of the musculoskeletal system and connective tissue	M76	1.93	1.89	1.98	$<1.0 \times 10^{-13}$
Diseases of the musculoskeletal system and connective tissue	M77	1.97	1.95	1.99	$<1.0 \times 10^{-13}$
Diseases of the musculoskeletal system and connective tissue	M79	2.59	2.57	2.62	$<1.0 \times 10^{-13}$
Diseases of the musculoskeletal system and connective tissue	M80	2.49	2.45	2.53	$<1.0 \times 10^{-13}$
Diseases of the musculoskeletal system and connective tissue	M81	2.37	2.35	2.38	$<1.0 \times 10^{-13}$
Diseases of the musculoskeletal system and connective tissue	M82	2.88	2.65	3.13	$<1.0 \times 10^{-13}$
Diseases of the musculoskeletal system and connective tissue	M83	2.76	2.50	3.05	$<1.0 \times 10^{-13}$
Diseases of the musculoskeletal system and connective tissue	M84	2.39	2.27	2.53	$<1.0 \times 10^{-13}$

Disease group	ICD-10 code	Odds ratio	Lower 95% confidence interval	Upper 95% confidence interval	Bonferroni corrected p value
Diseases of the musculoskeletal system and connective tissue	M85	2.19	2.09	2.29	$<1.0 \times 10^{-13}$
Diseases of the musculoskeletal system and connective tissue	M86	2.04	1.92	2.17	$<1.0 \times 10^{-13}$
Diseases of the musculoskeletal system and connective tissue	M87	2.41	2.30	2.52	$<1.0 \times 10^{-13}$
Diseases of the musculoskeletal system and connective tissue	M88	1.61	1.37	1.88	3.32×10^{-6}
Diseases of the musculoskeletal system and connective tissue	M89	2.11	2.03	2.18	$<1.0 \times 10^{-13}$
Diseases of the musculoskeletal system and connective tissue	M90	2.25	1.87	2.71	$<1.0 \times 10^{-13}$
Diseases of the musculoskeletal system and connective tissue	M91	3.00	1.98	4.54	0.0002
Diseases of the musculoskeletal system and connective tissue	M92	2.13	1.83	2.48	$<1.0 \times 10^{-13}$
Diseases of the musculoskeletal system and connective tissue	M93	2.25	2.21	2.29	$<1.0 \times 10^{-13}$
Diseases of the musculoskeletal system and connective tissue	M94	2.12	2.07	2.18	$<1.0 \times 10^{-13}$
Diseases of the musculoskeletal system and connective tissue	M95	1.77	1.72	1.82	$<1.0 \times 10^{-13}$
Diseases of the musculoskeletal system and connective tissue	M96	2.77	2.68	2.86	$<1.0 \times 10^{-13}$
Diseases of the musculoskeletal system and connective tissue	M99	2.11	2.08	2.13	$<1.0 \times 10^{-13}$
Diseases of the genitourinary system	N00	2.25	1.95	2.60	$<1.0 \times 10^{-13}$
Diseases of the genitourinary system	N01	1.59	1.20	2.11	1
Diseases of the genitourinary system	N02	1.60	1.49	1.71	$<1.0 \times 10^{-13}$
Diseases of the genitourinary system	N03	1.84	1.71	1.98	$<1.0 \times 10^{-13}$
Diseases of the genitourinary system	N04	1.98	1.83	2.14	$<1.0 \times 10^{-13}$
Diseases of the genitourinary system	N05	2.00	1.88	2.12	$<1.0 \times 10^{-13}$
Diseases of the genitourinary system	N06	1.92	1.66	2.22	$<1.0 \times 10^{-13}$
Diseases of the genitourinary system	N07	1.87	1.46	2.40	0.001
Diseases of the genitourinary system	N08	1.85	1.82	1.88	$<1.0 \times 10^{-13}$
Diseases of the genitourinary system	N10	2.49	2.22	2.79	$<1.0 \times 10^{-13}$
Diseases of the genitourinary system	N11	1.90	1.81	1.98	$<1.0 \times 10^{-13}$
Diseases of the genitourinary system	N12	2.33	2.20	2.47	$<1.0 \times 10^{-13}$
Diseases of the genitourinary system	N13	1.47	1.42	1.52	$<1.0 \times 10^{-13}$
Diseases of the genitourinary system	N14	2.39	2.13	2.69	$<1.0 \times 10^{-13}$
Diseases of the genitourinary system	N15	2.17	1.79	2.63	4.71×10^{-12}
Diseases of the genitourinary system	N16	2.40	1.96	2.93	$<1.0 \times 10^{-13}$
Diseases of the genitourinary system	N17	2.46	2.31	2.62	$<1.0 \times 10^{-13}$
Diseases of the genitourinary system	N18	1.89	1.88	1.91	$<1.0 \times 10^{-13}$
Diseases of the genitourinary system	N19	2.17	2.14	2.20	$<1.0 \times 10^{-13}$
Diseases of the genitourinary system	N20	1.59	1.56	1.61	$<1.0 \times 10^{-13}$
Diseases of the genitourinary system	N21	1.39	1.26	1.53	2.48×10^{-8}
Diseases of the genitourinary system	N22	1.57	1.00	2.47	1
Diseases of the genitourinary system	N23	1.90	1.73	2.09	$<1.0 \times 10^{-13}$
Diseases of the genitourinary system	N25	1.89	1.82	1.95	$<1.0 \times 10^{-13}$
Diseases of the genitourinary system	N26	1.83	1.76	1.89	$<1.0 \times 10^{-13}$
Diseases of the genitourinary system	N27	2.00	1.77	2.25	$<1.0 \times 10^{-13}$

Disease group	ICD-10 code	Odds ratio	Lower 95% confidence interval	Upper 95% confidence interval	Bonferroni corrected p value
Diseases of the genitourinary system	N28	1.65	1.63	1.67	$<1.0 \times 10^{-13}$
Diseases of the genitourinary system	N29	1.89	1.58	2.28	1.04×10^{-8}
Diseases of the genitourinary system	N30	1.81	1.77	1.85	$<1.0 \times 10^{-13}$
Diseases of the genitourinary system	N31	1.87	1.83	1.90	$<1.0 \times 10^{-13}$
Diseases of the genitourinary system	N32	1.94	1.90	1.99	$<1.0 \times 10^{-13}$
Diseases of the genitourinary system	N33	2.25	1.87	2.71	$<1.0 \times 10^{-13}$
Diseases of the genitourinary system	N34	2.26	2.07	2.46	$<1.0 \times 10^{-13}$
Diseases of the genitourinary system	N35	1.95	1.88	2.02	$<1.0 \times 10^{-13}$
Diseases of the genitourinary system	N36	1.85	1.68	2.02	$<1.0 \times 10^{-13}$
Diseases of the genitourinary system	N37	3.81	1.88	7.71	0.225
Diseases of the genitourinary system	N39	1.90	1.88	1.92	$<1.0 \times 10^{-13}$
Diseases of the genitourinary system	N40	1.55	1.54	1.56	$<1.0 \times 10^{-13}$
Diseases of the genitourinary system	N41	1.85	1.79	1.91	$<1.0 \times 10^{-13}$
Diseases of the genitourinary system	N42	1.62	1.57	1.68	$<1.0 \times 10^{-13}$
Diseases of the genitourinary system	N43	1.53	1.48	1.58	$<1.0 \times 10^{-13}$
Diseases of the genitourinary system	N44	2.14	1.32	3.49	1
Diseases of the genitourinary system	N45	1.98	1.85	2.12	$<1.0 \times 10^{-13}$
Diseases of the genitourinary system	N46	1.62	1.43	1.85	2.28×10^{-10}
Diseases of the genitourinary system	N47	1.57	1.49	1.65	$<1.0 \times 10^{-13}$
Diseases of the genitourinary system	N48	1.70	1.65	1.76	$<1.0 \times 10^{-13}$
Diseases of the genitourinary system	N49	1.99	1.67	2.38	3.37×10^{-11}
Diseases of the genitourinary system	N50	1.86	1.77	1.96	$<1.0 \times 10^{-13}$
Diseases of the genitourinary system	N51	1.80	1.41	2.30	0.003
Diseases of the genitourinary system	N60	1.22	1.17	1.26	$<1.0 \times 10^{-13}$
Diseases of the genitourinary system	N61	2.62	2.29	2.99	$<1.0 \times 10^{-13}$
Diseases of the genitourinary system	N62	2.14	2.05	2.24	$<1.0 \times 10^{-13}$
Diseases of the genitourinary system	N63	1.64	1.48	1.82	$<1.0 \times 10^{-13}$
Diseases of the genitourinary system	N64	1.39	1.35	1.43	$<1.0 \times 10^{-13}$
Diseases of the genitourinary system	N70	2.00	1.75	2.30	$<1.0 \times 10^{-13}$
Diseases of the genitourinary system	N71	2.15	1.76	2.63	1.17×10^{-10}
Diseases of the genitourinary system	N72	1.20	1.03	1.40	1
Diseases of the genitourinary system	N73	2.05	1.90	2.21	$<1.0 \times 10^{-13}$
Diseases of the genitourinary system	N74	0.50	0.07	3.75	1
Diseases of the genitourinary system	N75	1.70	1.29	2.25	0.201
Diseases of the genitourinary system	N76	1.35	1.31	1.39	$<1.0 \times 10^{-13}$
Diseases of the genitourinary system	N77	1.81	1.62	2.01	$<1.0 \times 10^{-13}$
Diseases of the genitourinary system	N80	1.61	1.52	1.71	$<1.0 \times 10^{-13}$
Diseases of the genitourinary system	N81	1.48	1.45	1.51	$<1.0 \times 10^{-13}$
Diseases of the genitourinary system	N82	1.50	1.23	1.84	0.093
Diseases of the genitourinary system	N83	1.54	1.48	1.60	$<1.0 \times 10^{-13}$

Disease group	ICD-10 code	Odds ratio	Lower 95% confidence interval	Upper 95% confidence interval	Bonferroni corrected p value
Diseases of the genitourinary system	N84	1.08	1.00	1.18	1
Diseases of the genitourinary system	N85	1.15	1.09	1.20	1.09×10^{-5}
Diseases of the genitourinary system	N86	0.94	0.88	1.01	1
Diseases of the genitourinary system	N87	1.21	1.11	1.32	0.010
Diseases of the genitourinary system	N88	1.14	1.05	1.23	1
Diseases of the genitourinary system	N89	1.09	1.07	1.11	$<1.0 \times 10^{-13}$
Diseases of the genitourinary system	N90	1.33	1.27	1.40	$<1.0 \times 10^{-13}$
Diseases of the genitourinary system	N91	1.14	1.06	1.22	0.344
Diseases of the genitourinary system	N92	1.14	1.10	1.19	2.12×10^{-8}
Diseases of the genitourinary system	N93	1.26	1.15	1.39	0.001
Diseases of the genitourinary system	N94	1.21	1.16	1.26	$<1.0 \times 10^{-13}$
Diseases of the genitourinary system	N95	1.35	1.34	1.36	$<1.0 \times 10^{-13}$
Diseases of the genitourinary system	N96	0.17	0.02	1.23	1
Diseases of the genitourinary system	N97	1.21	1.04	1.42	1
Diseases of the genitourinary system	N98	1.29	0.38	4.31	1
Diseases of the genitourinary system	N99	1.78	1.70	1.85	$<1.0 \times 10^{-13}$

* Control group was matched by sex, age and associated regional ASHIP with a case-to-control-ratio of 1:9.
